# *O*-GlcNAcylation inhibition redirects the response of colon cancer cells to chemotherapy from senescence to apoptosis

**DOI:** 10.1038/s41419-024-07131-5

**Published:** 2024-10-19

**Authors:** Ingrid Loison, Adrien Pioger, Sonia Paget, Inès Metatla, OrgaRES Consortium, Christophe Mariette, Guillaume Piessen, François Corfiotti, Clarisse Eveno, François-René Pruvot, Stéphanie Truant, Mehdi El Amrani, Emmanuelle Leteurtre, Florence Renaud, Charlotte Dufour, Viviane Gnemmi, Laurence Wicquart, Fabienne Escande, Julie Leclerc, Isabelle Van Seuningen, Audrey Vincent, Corinne Abbadie, Vanessa Dehennaut

**Affiliations:** 1 University Lille, CNRS, Inserm, Institut Pasteur de Lille, CHU Lille, UMR9020-U1277 – CANTHER – Cancer Heterogeneity, Plasticity and Resistance to Therapies, F-59000 Lille, France; 2 University Lille, CNRS, OrgaLille Platform, F-59000 Lille, France; 3 Present Address: Proteomics Platform Necker, Université Paris Cité-Structure Fédérative de Recherche Necker, INSERM US24/CNRS UAR3633, 75015 Paris, France

**Keywords:** Cancer therapeutic resistance, Cancer metabolism

## Abstract

The potential use of pro-senescence therapies, known as TIS (Therapy-Induced Senescence), for the treatment of colorectal cancer (CRC) generated significant interest since they require lower doses compared to those required for inducing apoptosis. However, the senescent cell cycle-arrested cancer cells are long-lived, and studies have revealed escape mechanisms contributing to tumor recurrence. To deepen our understanding of the survival pathways used by senescent cancer cells, we delved into the potential involvement of the hexosamine biosynthetic pathway (HBP). HBP provides UDP-GlcNAc, the substrate for *O*-GlcNAc transferase (OGT), which catalyzes *O*-GlcNAcylation, a post-translational modification implicated in regulating numerous cellular functions and aberrantly elevated in CRC. In this study, we demonstrated, in the p53-proficient colon cancer cell lines HCT116 and LS174T, that TIS induced by low-dose SN38 or etoposide treatment was accompanied with a decrease of GFAT (the rate limiting enzyme of the HBP), OGT and *O*-GlcNAcase (OGA) expression correlated with a slight reduction in *O*-GlcNAcylation levels. Further decreasing this level of *O*-GlcNAcylation by knocking-down GFAT or OGT redirected the cellular response to subtoxic chemotherapy doses from senescence to apoptosis, in correlation with an enhancement of DNA damages. Pharmacological inhibition of OGT with OSMI-4 in HCT116 and LS174T cells and in a patient-derived colon tumoroid model supported these findings. Taken together, these results suggest that combing *O*-GlcNAcylation inhibitors to low doses of conventional chemotherapeutic drugs could potentially reduce treatment side effects while preserving efficacy. Furthermore, this approach may increase treatment specificity, as CRC cells exhibit higher *O*-GlcNAcylation levels compared to normal tissues.

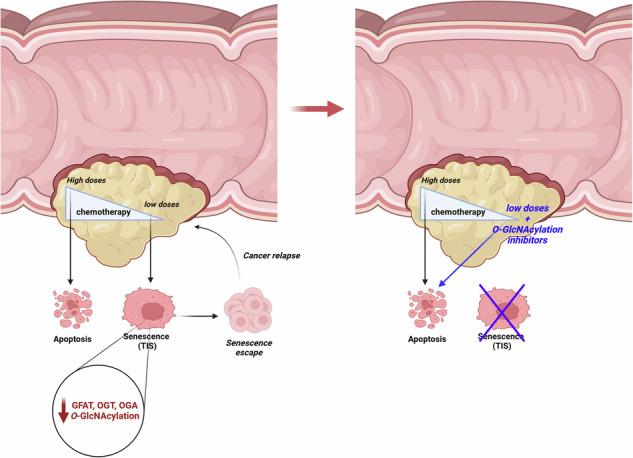

## Introduction

With more than one million diagnosed cases worldwide in 2020, colorectal cancer (CRC) ranks as the third most common cancer and the second leading cause of cancer-related deaths, regardless of gender [1]. Currently, combination of surgery and chemotherapy remains the common treatment for CRC. Thanks to the refinement and diversification of the therapeutic protocols, the overall survival of patients with advanced CRC has shown improvement over recent decades. Nevertheless, even though the response rate to current systemic chemotherapies can reach up to 50%, nearly all patients develop drug resistance, resulting in disease relapse and diminished overall survival [2]. An increasing number of studies tend to show that senescence could be one of the mechanisms responsible for cancer cells’ resistance to chemotherapy and tumor recurrence [3].

Originally described as the state reached by normal human fibroblasts after a finite number of doublings, senescence is now recognized as a stress response program that can be activated following telomeric shortening, accumulation of DNA damage, oxidative stress, or even oncogene activation. The senescence program includes a prolonged cell cycle arrest mainly mediated by the p53/p21 and/or p16/RB pathways, a chromatin reorganization, a specific secretome called SASP (Senescence Associated Secretory Phenotype), a resistance to apoptosis, and a reprogrammed metabolism [4]. Many studies have also shown that senescence can be induced in normal and cancer cells as well by various genotoxic agents used in anticancer therapy; this senescence is called “Therapy-Induced Senescence” (TIS) [5, 6]. While first considered as a curative outcome since inducing a stable proliferation arrest in cancer cells, several studies, notably conducted in the context of CRC, have reported the existence of TIS-escape mechanisms during which a few senescent cancer cells are capable of re-entering the cell cycle. Importantly, cells that have escaped TIS can have acquired stem cell characteristics and an even more transformed phenotype and therefore might be responsible for recurrence of a cancer more aggressive than the initial one [7, 8] [3, 9–13].

While reprogramming of energy metabolism has been extensively explored in the context of oncogenesis and is a well-recognized hallmark of cancer cells [14], the importance of glycolysis and other aspects of metabolism has only recently begun to be studied in the context of anti-tumor responses such as TIS. These studies have nevertheless revealed the key role of several aspects of cellular metabolism, especially glycolysis and glutaminolysis, in the establishment and control of senescent phenotypes, and that modulation of the expression of certain metabolic enzymes disrupts the response of cancer cells to TIS [8, 15–18].

**Fig. 1:**
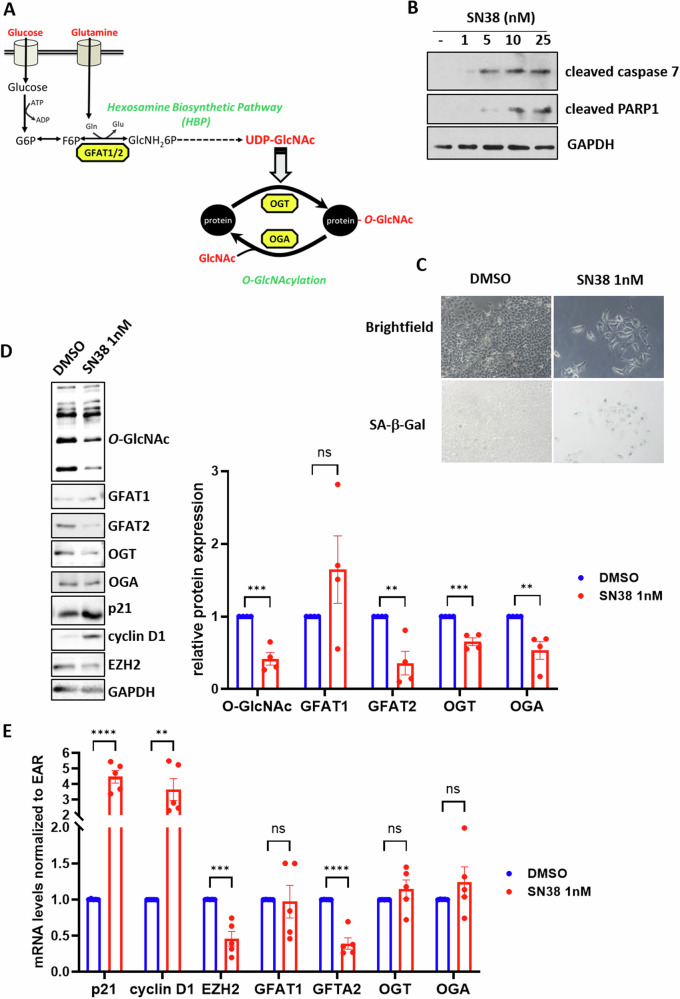
SN38-induced senescence of HCT116 cells is accompanied by a decrease in GFAT2, OGT, OGA, and *O*-GlcNAcylation levels. **A** Scheme depicting the Hexosamine Biosynthetic Pathway and *O*-GlcNAcylation processes. GFAT1/2: Glutamine Fructose-6-Phosphate Amido Transferase 1/2 (rate limiting enzyme of the HBP), OGT: *O*-GlcNAc Transferase, OGA: *O*-GlcNAcase. **B** HCT116 cells were treated with increasing concentrations of SN38 ranging from 1 to 25 nM for 96 hours. Cell apoptosis was investigated by Western Blot analyses of cleaved-caspase 7 and cleaved-PARP1. GAPDH was used as a loading control. Data shown are representative of three independent experiments. **C–E** HCT116 cells were treated with 1 nM SN38 for 96 or with DMSO as a negative control. **C** Top: Representative images of the microscopic analysis of cell morphology showing an increase in the size of senescent cells. Pictures were taken at the same magnification (x200). Bottom: SA-β-Galactosidase activity assay demonstrating blue staining of senescent cells. **D** Left: Western blot analysis of *O*-GlcNAcylation levels, OGT, OGA, and both isoforms of GFAT (GFAT1 and GFAT2), as well as the expression of p21, cyclin D1, and EZH2, three senescence markers. Right: Quantification of relative protein expression from four independent experiments (optical density measurement relative to GAPDH) (*n* = 4) (individual values and mean +/- SEM, ns: non-significant, *P < 0.05, **P < 0.01, multiple unpaired *t*-tests). **E** qRT-PCR analysis of p21, cyclin D1, EZH2, GFAT1, GFAT2, OGT, and OGA transcript expression. Results represent the individual values and mean +/- SEM of five independent experiments (*n* = 5) (ns: non-significant, **P < 0.01, ***P < 0.001, ****P < 0.0001, multiple unpaired *t*-tests).

Among the metabolic pathways that also use glucose and glutamine, the hexosamine biosynthesis pathway (HBP) supplies UDP-GlcNAc (Fig. 1A). This sugar-nucleotide is required for the biosynthesis of many membrane glycans, but is also the substrate for *O*-N-Acetylglucosaminyl (*O*-GlcNAc) Transferase (OGT). This enzyme ensures the transfer of a GlcNAc residue onto Serines or Threonines of its thousands of target proteins and is the only glycosyltransferase capable of post-translationally modifying cytosolic and nuclear proteins [19]. *O*-GlcNAcylation of nucleocytoplasmic proteins is dynamic and reversible: *O*-GlcNAcase (OGA) catalyzes the hydrolysis of the GlcNAc residue.

Numerous studies have shown a link between tumor development and increased glucose flux through the HBP pathway, particularly in CRC [20]. Our previous work has also demonstrated an increase in *O*-GlcNAcylation levels and OGT expression in human CRC samples compared to normal tissues [21], as well as in a murine model of colon carcinogenesis [22]. Moreover, we have shown that OGT knockdown by siRNA reduced the proliferation, survival, and adhesion of colon cancer cells [23]. These works, among others, have contributed to define hyper-*O*-GlcNAcylation as a new hallmark of CRC and as a potential therapeutic target [24, 25].

In this study, we investigated whether the HBP and *O*-GlcNAcylation processes could regulate the entry into TIS of colon cancer cells.

## Results

### Entry of p53-proficient colon cancer cells into TIS is accompanied by a decrease of GFAT, OGT and OGA expression as well as O-GlcNAcylation levels

There are two major types of colorectal cancer. On one hand, there are CRC exhibiting a microsatellite instability phenotype with deficient mismatch repair (MSI/dMMR). On the other hand, there are CRC that do not exhibit microsatellite instability or mismatch repair deficiency but have chromosomal instability (MSS/CIN). MSI/dMMR CRC represent only 15% of localized CRC cases and 5% of metastatic CRC. At the metastatic stage they have a less favorable prognosis compared to MSS/CIN CRC [26, 27]. Interestingly, these two types of CRC have different mutational profiles, notably regarding the *TP53* tumor suppressor gene encoding the p53 protein. Indeed, this gene is frequently mutated in MSS/CIN CRC, whereas it is very rarely mutated in MSI/dMMR CRC. During TIS, cell proliferation arrest is principally mediated by the activation of the p53 pathway, and mutations in this pathway could render colon cancer cells insensitive to TIS. However, it has been shown that certain neoplastic cells retain the ability to enter senescence after various stresses, even in the absence of a functional *TP53* gene [5]. We therefore first aimed to study the expression of enzymes of the HBP pathway and *O*-GlcNAcylation processes (Fig. 1A) upon chemotherapy-induced senescence in various colon cancer cell lines with different p53 statuses: in two MSI/dMMR cell lines expressing the wild-type *TP53* gene: HCT116 and LS174T (Fig. 1, Supplementary Figs. S3 and S4) and in two MSS/CIN cell lines mutated for this gene: HT29 and Caco2 (Supplementary Fig. S5). In several cancer cell lines, it has been shown that treatment with low doses of chemotherapy triggers senescence while apoptosis is induced at higher concentrations suggesting that the senescence response only occurs in a specific range of DNA damage [28]. We thus first treated HCT116 colon cancer cells with various concentrations of SN8, the active form of irinotecan, a commonly used chemotherapeutic agent in CRC treatment. Ninety-six hours after the treatment, we observed apoptosis of the cells only at doses greater than 5 nM, as evidenced by the detection of cleaved forms of caspase 7 and PARP1 (Fig. 1B). At the concentration of 1 nM, we observed induction of senescence in the cells, as attested by the detection of several senescence markers: increased cell size and granularity (Fig. 1C), enhanced β-galactosidase activity (Fig. 1C), elevated p21 and cyclin D1 expression (indicative of cell cycle arrest) **(**Fig. 1D, E) and decreased expression of the epienzyme EZH2, characteristic of the epigenetic reprogramming occurring during TIS [9–11] (Fig. 1D, E). Moreover, HCT116 cells treated with 1 nM SN38 for 96 h were sensitive to the well-characterized senolytic navitoclax, whereas non-treating cells were not (Supplementary Fig. S1) further demonstrating that they were effectively in a senescent state. We also checked for the p53 dependency of this SN38-induced senescence. For that, HCT116 cells were transfected with a siRNA targeting p53 24 h prior to a 1 nM SN38 treatment (Supplementary Fig. S2A, B). In the sip53 -transfected cells, we did not observe, as expected, any increase in p21 expression (a direct targetgene of p53) in response to 1 nM SN38 treatment. Furthermore, we did not observe any increase in cyclin D1 expression, SA-β-galactosidase activity and any decrease in EZH2 expression. Rather, we observed the appearance of the apoptotic marker cleaved-PARP1 correlated with higher expression of γH2AX, a sensor of DNA damages. We also tried to induce senescence in HCT116 cells knocked-down for p53 by treating them with lower concentrations of SN38 ranging from 0.1 to 0.5 nM leading to less DNA damages but without success (Supplementary Fig. S2B and S2C). Collectively, these data demonstrate that the SN38-induced senescence in HCT116 cells rely on a functional p53 pathway.

In these senescent HCT116 cells, we analyzed the expression of GFAT1 and GFAT2 (the two isoforms of the rate-limiting enzyme of the HBP), OGT and OGA at the protein (Fig. 1C) and mRNA levels (Fig. 1D). Additionally, we examined the overall *O*-GlcNAcylation levels (Fig. 1D). We observed decreased expression of GFAT2, OGT and OGA, which correlated with reduced *O*-GlcNAcylation levels in SN38-induced senescent cells compared to proliferating control cells (Fig. 1D). The decrease in GFAT2 expression occurs at both the mRNA and protein levels while the reduction in OGT and OGA expression was observed only at the post-transcriptional level (Fig. 1E). Similar reprogramming of the HBP and *O*-GlcNAcylation processes were also observed in HCT116 cells induced into TIS with etoposide, another chemotherapeutic agent (Supplementary Fig. S3) and in LS174T colon cancer cells induced into TIS with either SN38 or etoposide (Supplementary Fig. S4). In LS174T cells, reduced *O*-GlcNAcylation levels were correlated with decreased protein expression of OGT, OGA and GFAT1, the only isoform of GFAT expressed in this cell line. These changes occurred primarily at the post-transcriptional level.

We then performed similar analyses in HT29 and Caco2 cells (Supplementary Fig. S5). In accordance with the literature, we observed that these p53-mutated cells appeared less susceptible to TIS induction, exhibiting a different phenotype compared to p53-proficient senescent cells; specifically, discrete changes in cell morphology, a modest increase in SA-β-galactosidase activity in response to subtoxic doses of SN38, and intriguingly, no obvious variation in cyclin D1 and EZH2 expression. In these cells, we did not observe any change in GFAT, OGT, or OGA expression, nor any variation in *O*-GlcNAcylation levels in response to SN38.

Collectively, these results strongly indicate that the reprogramming of the HBP and *O*-GlcNAcylation processes represent a consistent characteristic of p53-dependent TIS of colon cancer cells while it does not occur during p53-independent senescence.

### The decrease in O-GlcNAcylation levels is neither sufficient nor necessary for p53-proficient colon cancer cells to enter senescence

Next, we asked whether the reprogramming of the HBP and *O*-GlcNAcylation processes was essential for the occurrence of TIS in p53-proficient colon cancer cells. Knock-down experiments targeting either GFAT1 and GFAT2 alone or in combination revealed that reducing GFAT and consequently *O*-GlcNAcylation levels was not sufficient to induce senescence of HCT116 cells in the absence of a chemotherapeutic treatment (Supplementary Fig. S6). Additionally, reducing *O*-GlcNAcylation levels through the inhibition of OGT expression, using siRNA, or activity, with the pharmacological inhibitor OSMI-4, did not induce senescence by themselves (Supplementary Fig. S7) even though targeting OGT appears to induce cell cycle arrest, as evidenced by the upregulation of p21 and cyclin D1 expression.

**Fig. 2:**
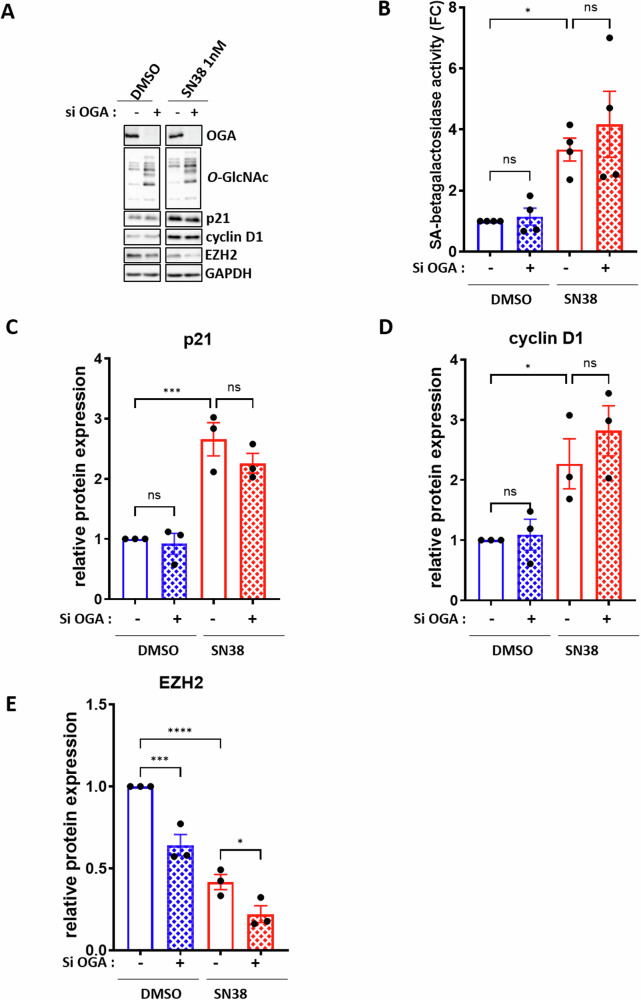
Preventing *O*-GlcNAcylation decrease through OGA silencing does not impact the entry of HCT116 cells into SN38-induced senescence. HCT116 cells were transfected with a siRNA targeting OGA (si OGA) or a non-target control siRNA (siCTRL). 24 h later, the cells were treated with 1 nM SN38 for 72 hours. **A** Western blot analysis was performed to assess the expression of the three senescence markers p21, cyclin D1, and EZH2. The efficiency of siRNA was also confirmed by evaluating OGA los of expression and O-GlcNAcylation levels upregulation. GAPDH was used as a loading control. Results shown are representative of three independent experiments. **B** Quantitative fluorimetric determination of the SA-β-Galactosidase activity in the different experimental conditions. Results represent the individual values and mean +/- SEM of four independent experiments (*n* = 4) (ns: non-significant, *P < 0.05, ordinary one-way ANOVA with Fisher’s LSD multiple comparison tests). Quantification of relative protein expression analyzed by Western Blot (optical density measurement relative to GAPDH) of p21 (**C**), cyclin D1 (**D**) and EZH2 (**E**) from three independent experiments (*n* = 3) (individual values and mean +/- SEM, ns: non-significant, *P < 0.05, ***P < 0.001, ****P < 0.0001, ordinary one-way ANOVA with Fisher’s LSD multiple comparison tests).

We then investigated whether the decrease in *O*-GlcNAcylation was essential for the entry of p53-proficient colon cancer cells into senescence in response to chemotherapy. To this end, we examined the consequences of maintaining *O*-GlcNAcylation at a high level, through OGA knockdown, on the entry of cells into TIS, hypothesizing that it could potentially delay or prevent the establishment of the senescence phenotype. To assess this, HCT116 cells were transfected with siRNA targeting OGA 24 hours prior to treatment with or without 1 nM SN38 for 72 h (Fig. 2). As expected, the *O*-GlcNAcylation levels remains high in siOGA-transfected cells (Fig. 2A), however it did not prevent the upregulation of SA-β-galactosidase activity (Fig. 2B), nor the induction of p21 and cyclin D1 expression (Fig. 2A, C, D), or the reduction of EZH2 expression (Fig. 2A, E) induced by the SN38 treatment. Similar results were obtained upon OGA knockdown combined with lowdose etoposide treatment (Supplementary Fig. S8).

Taken together, these results demonstrate that the decrease in *O*-GlcNAcylation levels is neither sufficient nor necessary for p53-proficient colon cancer cells to enter senescence.

### Inhibition of GFAT or OGT combined with a low-dose chemotherapy treatment induces a switch of p53-proficient colon cancer cells from senescence to apoptosis

**Fig. 3:**
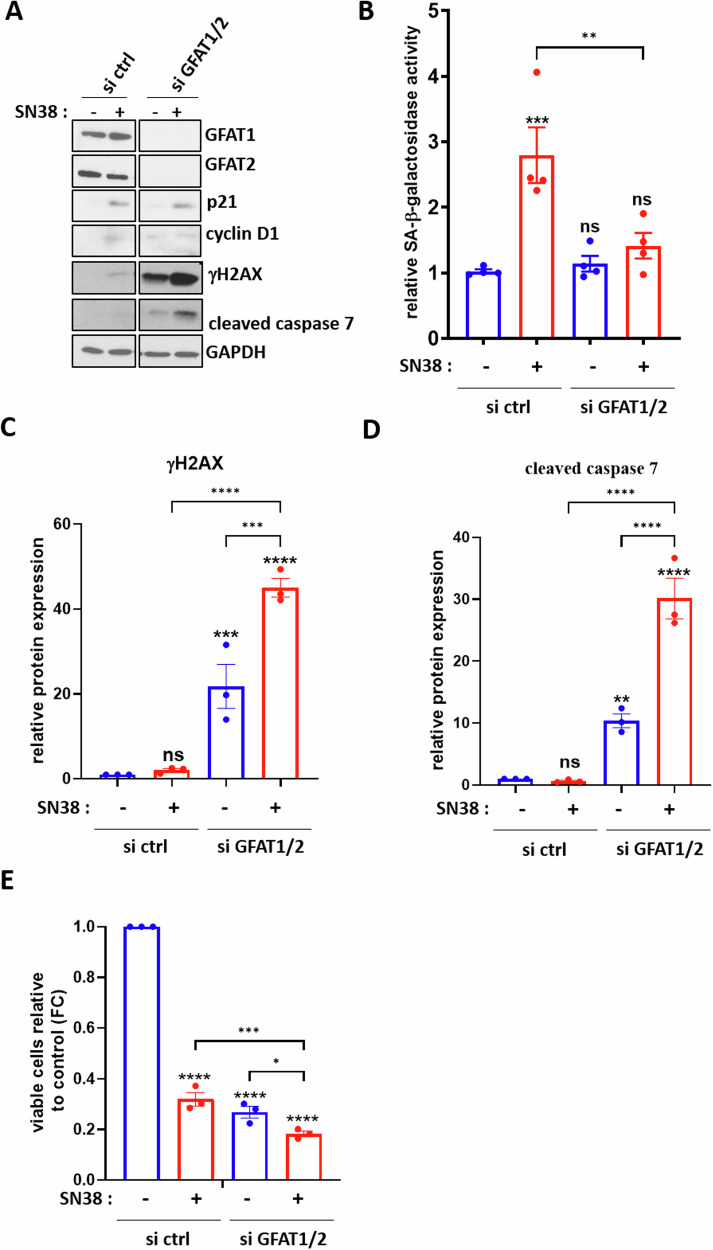
Silencing GFAT1 and GFAT2 prevents the entry of HCT116 cells into SN38-induced senescence and induces their apoptosis through enhancement of DNA damage. HCT116 cells were transfected with a siRNA targeting GFAT1 and GFAT2 (si GFAT1/2) or a non-target control siRNA (siCTRL). 24 h later, the cells were treated with 1 nM SN38 for 72 h. **A** Western blot analysis was performed to assess the expression of the senescence markers p21 and cyclin D1, the DNA damage marker γH2AX and the apoptosis marker cleaved-caspase 7. The efficiency of siRNA was also confirmed by evaluating GFAT1 and GFAT2 loss of expression. GAPDH was used as a loading control. Results shown are representative of three independent experiments. **B** Quantitative fluorimetric determination of the SA-β-Galactosidase activity in the different experimental conditions. Results represent the individual values and mean +/- SEM of four independent experiments (*n* = 4) (ns: non-significant, **P < 0.01, ***P < 0.001, ordinary one-way ANOVA with Fisher’s LSD multiple comparison tests). Quantification of relative protein expression analyzed by Western Blot (optical density measurement relative to GAPDH) of γH2AX (**C**) and cleaved-caspase 7 (**D**) from three independent experiments (*n* = 3) (individual values and mean +/- SEM, ns: non-significant, *P < 0.05, **P < 0.01, ***P < 0.001, ordinary one-way ANOVA with Fisher’s LSD multiple comparison tests). **E** Quantification of the ratio of viable cells through MTT assay from three independent experiments (*n* = 3) (individual values and mean +/- SEM, ****P < 0.0001, ***P < 0.001, *P < 0.05, ordinary one-way ANOVA with Fisher’s LSD multiple comparison tests).

We last explored whether induction of a decrease in *O*-GlcNAcylation through GFAT or OGT inhibition could potentiate the entry of HCT116 cells into senescence in response to chemotherapy. To address this, cells were transfected with siRNA targeting GFAT1 and GFAT2 (si GFAT1/2) for 24 h before being treated or not with 1 nM SN38 for 72 h (Fig. 3). In cells transfected with a non-targeting scrambled siRNA (si ctrl) and treated with SN38, we observed, as expected, increased expression of cyclin D1 and p21 (Fig. 3A) and enhanced SA-β-galactosidase activity (Fig. 3B) compared to non-treated cells, indicating induction of senescence under this condition. Unexpectedly, this SN38-induced senescence was not observed in cells transfected with siRNA against GFAT1/2.

As mentioned above, previous studies have suggested that senescence induction in response to chemotherapy occurs within a specific range of DNA damage. Therefore, we analyzed the expression of the γ phosphorylated form of the histone H2AX (γH2AX), a sensor of DNA damage and a key regulator of the DDR (Fig. 3A, C), the cleaved form of caspase 7, a marker of apoptosis (Fig. 3A, D) and performed a cell viability assay (Fig. 3E). While the number of viable control cells strongly decreases in response to SN38 treatment, this decrease is associated with only a slight increase in γH2AX expression and no caspase 7 cleavage. This demonstrates that the reduction in the number of viable cells is related to the cell cycle arrest occurring during TIS.

siGFAT1/2 transfection in non-treated cells also led to a decreased number of viable cells but was clearly associated with γH2AX overexpression and caspase 7 cleavage, indicating apoptosis of the cells. Furthermore, this increase in γH2AX and cleaved-caspase 7 correlated with a diminution of cell viability was even greater in cells invalidated for GFAT1/2 and further treated with SN38. This last result indicates that inhibiting GFAT1/2 in combination with a low-dose SN38 treatment induces a switch of HCT116 cells from senescence to apoptosis. This decrease in senescence induction correlated with an increase in DNA damage and apoptosis was also observed in HCT116 cells upon GFAT1/2 knockdown combined with low-dose etoposide treatment (Supplementary Fig. S9).

**Fig. 4:**
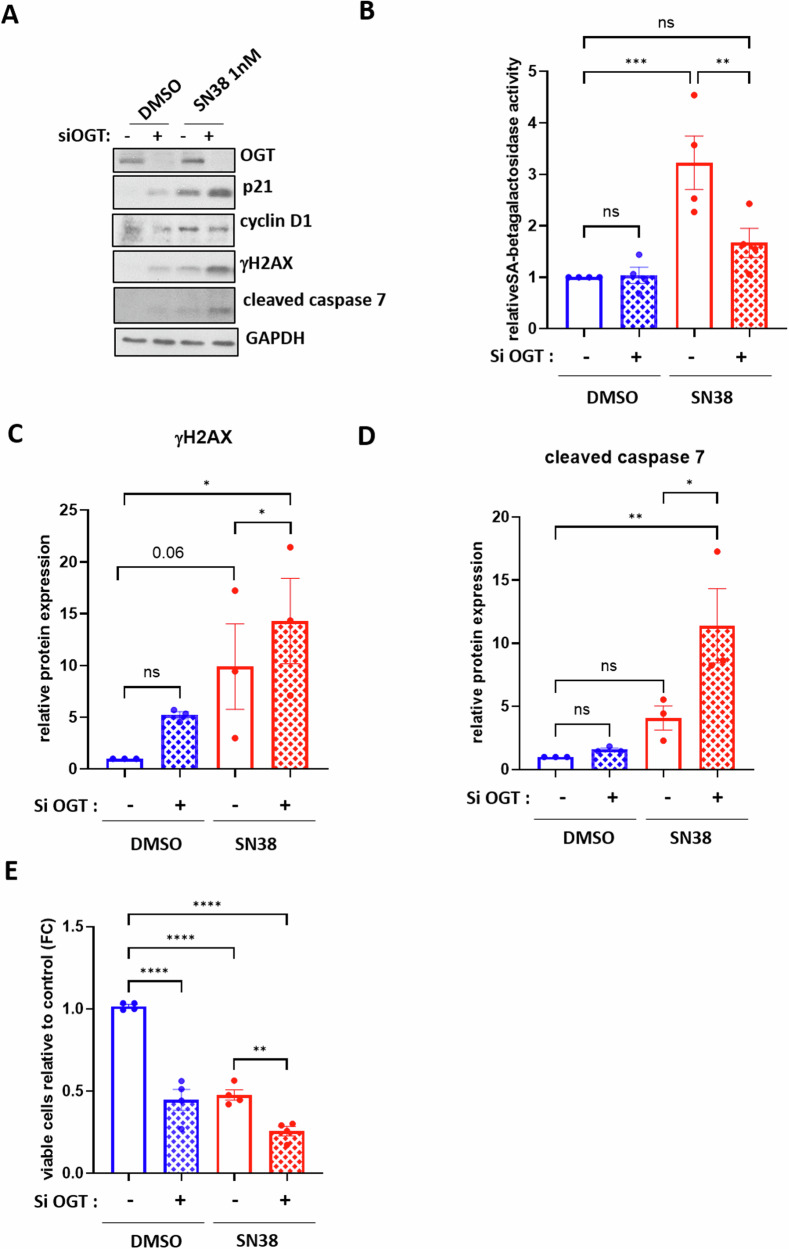
Silencing OGT prevents the entry of HCT116 cells into SN38-induced senescence and induces their apoptosis through enhancement of DNA damage. HCT116 cells were transfected with a siRNA targeting OGT (si OGT) or a non-target control siRNA (siCTRL). 24 h later, the cells were treated with 1 nM SN38 for 72 h. **A** Western blot analysis was performed to assess the expression of the senescence markers p21 and cyclin D1, the DNA damage marker γH2AX and the apoptosis marker cleaved-caspase 7. The efficiency of siRNA was also confirmed by evaluating GFAT1 and GFAT2 loss of expression. GAPDH was used as a loading control. Results shown are representative of four independent experiments. **B** Quantitative fluorimetric determination of the SA-β-Galactosidase activity in the different experimental conditions. Results represent the individual values and mean +/- SEM of four independent experiments (*n* = 4) (ns: non-significant, **P < 0.01, ***P < 0.001, ordinary one-way ANOVA with Fisher’s LSD multiple comparison tests). Quantification of relative protein expression analyzed by Western Blot (optical density measurement relative to GAPDH) of γH2AX **(C)** and cleaved-caspase 7 **(D)** from three independent experiments (*n* = 3) (individual values and mean +/- SEM, ns: non-significant, *P < 0.05, **P < 0.01, ordinary one-way ANOVA with Fisher’s LSD multiple comparison tests). **E** Quantification of the ratio of viable cells through MTT assay from four independent experiments (n = 4) (mean +/- SEM, **P < 0.01, ****P < 0.0001, ordinary one-way ANOVA with Fisher’s LSD multiple comparison tests).

We then conducted similar experiments, this time targeting OGT prior to treatment with a low dose of either SN38 (Fig. 4) or etoposide (Supplementary Fig. S10). As already observed in Supplementary Fig. S7, targeting OGT in the absence of chemotherapy does not affect SA-β-galactosidase activity (Fig. 4B and Supplementary Fig. S10B). However, it leads to increased expression of p21, cyclin D1, and γH2AX (Fig. 4A, C and Supplementary Fig. S10A), along with a 50% reduction in viable cell count (Fig. 4E and Supplementary Fig. S10C), despite no induction of caspase 7 cleavage (Fig. 4D and Supplementary Fig. S10A). Again, this indicates that siOGT alone induces a cell cycle arrest. Similarly to siGFAT, we once again observed a decrease in SA-β-galactosidase activity (Fig. 4B and Supplementary Fig. S10B) along with an increase in DNA damage (Fig. 4A, C and Supplementary Fig. S10A) and induction of cell death (Fig. 4A, D, E and supplementary Fig. S10A and S10C) in cells transfected with siRNA targeting OGT and treated with a subtoxic dose of the chemotherapeutic agent compared to cells treated with the chemotherapy alone.

**Fig. 5:**
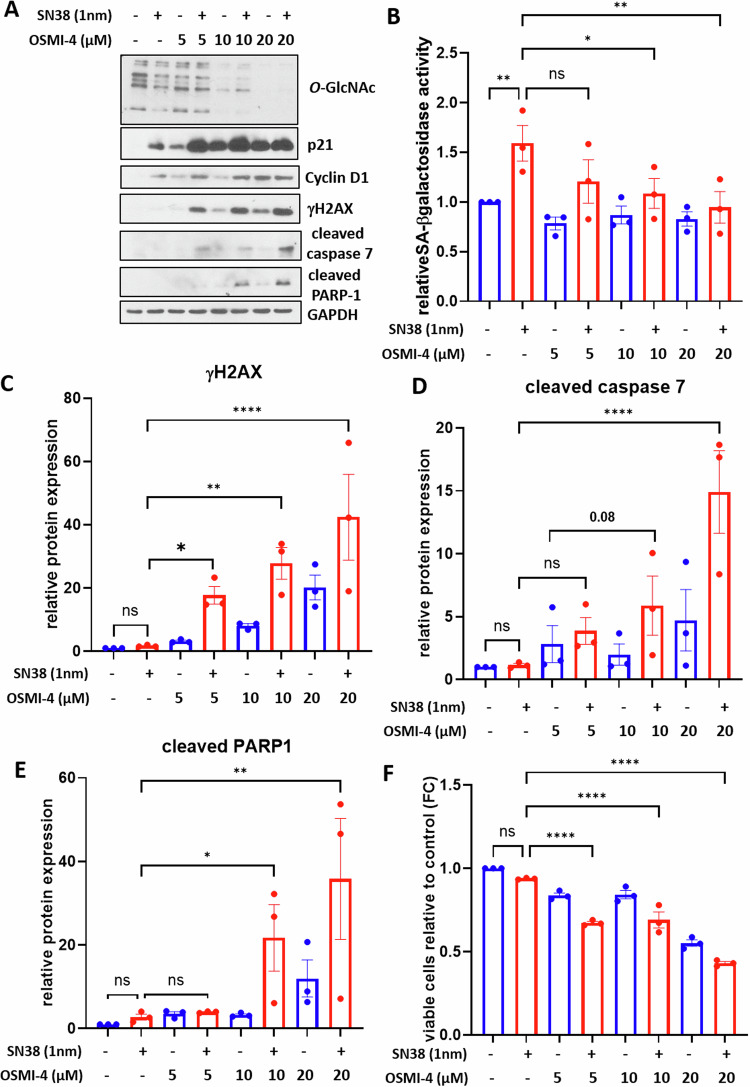
Pharmacological inhibition of OGT combined with low-dose SN38 treatment causes HCT116 cells to switch from senescence to apoptosis in a dose dependent-manner. HCT116 cells were treated with increasing concentrations of the OGT inhibitor OSMI-4 ranging from 5 to 20 µM. 24 h later, the cells were treated with 1 nM SN38 for 72 h. **A** Western blot analysis was performed to assess the expression of the senescence markers p21 and cyclin D1, the DNA damage marker γH2AX and the apoptosis markers cleaved-caspase 7 and cleaved-PARP1. The treatment efficiency was also confirmed by evaluating *O*-GlcNAcylation levels. GAPDH was used as a loading control. Results shown are representative of three independent experiments. **B** Quantitative fluorimetric determination of the SA-β-Galactosidase activity in the different experimental conditions. Results represent the individual values and mean +/- SEM of three independent experiments (*n* = 3) (ns: non-significant, *P < 0.05, **P < 0.01, ordinary one-way ANOVA with Fisher’s LSD multiple comparison tests). Quantification of relative protein expression analyzed by Western Blot (optical density measurement relative to GAPDH) of γH2AX (**C**) cleaved-caspase 7 (**D**) and cleaved-PARP1 (**E**) from three independent experiments (*n* = 3) (individual values and mean +/- SEM, ns: non-significant, *P < 0.05, **P < 0.01, ****P < 0.0001, ordinary one-way ANOVA with Fisher’s LSD multiple comparison tests). **F** Quantification of the ratio of viable cells through MTT assay from three independent experiments (*n* = 3) (mean +/- SEM, ***P < 0.001, ****P < 0.0001, ordinary one-way ANOVA with Fisher’s LSD multiple comparison tests).

To validate these findings, we treated HCT116 cells with increasing concentrations of the highly specific OGT inhibitor OSMI-4 in combination with 1 nM SN38 treatment (Fig. 5). Consistent with the siOGT experiments, OSMI-4 alone induced a cell cycle arrest. However, when used in conjunction with a low dose of SN38, OSMI-4 resulted in a dose-dependent decrease in *O*-GlcNAcylation levels (Fig. 5A) and SA-β-galactosidase activity compared to cells treated with SN38 alone (Fig. 5B). This combination also led to a dose-dependent increase in γH2AX (Fig. 5A, C), cleaved caspase 7 (Fig. 5A, D), and cleaved PARP-1 expression (Fig. 5A, E), as well as a dose-dependent decrease in cell viability (Fig. 5F).

We obtained the same results in the LS174T cell line treated with SN38 in combination with OSMI-4 (Supplementary Fig. S11). Thus collectively, our results demonstrate that inhibition of GFAT or OGT combined with low-dose chemotherapy treatment induces a switch of colon cancer cells from senescence to apoptosis associated with an enhancement of DNA damage. Therefore, we hypothesized that targeting *O*-GlcNAcylation in combination with chemotherapy could be a promising synthetic lethality approach for MSI/dMMR/p53 unmutated CRC treatment, abolishing the risk of senescence escape and cancer relapse.

### OGT inhibition enhances the cytotoxicity of SN38 in patient-derived colon tumor organoids

**Fig. 6:**
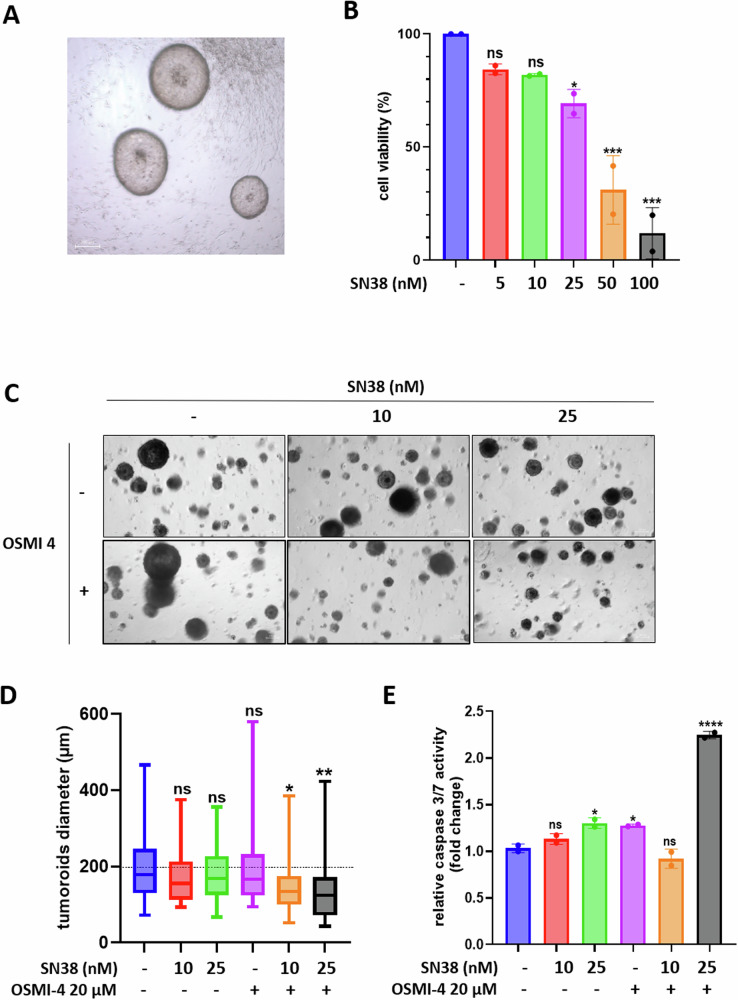
The OGT inhibitor OSMI-4 enhances the cytotoxicity of SN38 in patient-derived colon tumor organoids. **A** PDTOs derived from a human left colon tumor were allowed to form before being treated with increasing concentrations of SN38 ranging from 5 to 100 nM for 72 h. **B** Cell viability was evaluated through cell-titer glo® assay. Values represent the mean +/- SD of two independent experiments (*n* = 2) (ns: non-significant, *P < 0.05, ****P < 0.0001, ordinary one-way ANOVA with Dunnett’s multiple comparisons tests). **C**,**D** PDTOs were allowed to form before being treated either with 10 nM or 25 nM of SN38 alone or in combination with 20 µM OSMI-4 for five days. **C** Representative brightfield images of the tumoroïds in the different experimental conditions. Scale bar = 200 µM. **D** Box-plots depicting the diameters (in µM) of more than 30 tumoroïds obtained from three independent images acquired in one experiment and measured with the image J software. Statistical significance was evaluated using ordinary one-Way ANOVA with Tukey’s multiple comparisons tests (ns: non-significant, *P < 0.05, **P < 0.01). **E** Evaluation of apoptosis through caspase-glo® 3/7 activity assay. Values represent the individual values and mean +/- SEM of two replicates (ns: non-significant, *P < 0.05, ****P < 0.0001, ordinary one-way ANOVA with Dunnett’s multiple comparisons tests).

To evaluate the potential clinical value of a combined *O*-GlcNAcylation inhibition with chemotherapy for the treatment of CRC, we used patient-derived tumor organoïds (PDTOs). PDTOs offer the advantage of closely resembling the original tumor in terms of phenotype, molecular profile, and histology, and have demonstrated significant potential in predicting clinical responses to CRC therapy [29]. We generated PDTOs from a 76-year-old female with a well-differentiated pT3N0, Grade IIA sigmoid colon cancer and naive from chemotherapy and called them LeCo-2920vi (Fig. 6A).

In a first set of experiments, we evaluated the sensitivity of the LeCo-2920vi tumoroïds to SN38. For that, we treated them with increasing concentrations of SN38 ranging from 5 to 100 nM for 72 hours and measured cell viability (Fig. 6B). The results showed no statistically significant cell death when the PDTOs were treated with 5 or 10 nM SN38. However, a 30% decrease in cell viability was observed upon treatment with 25 nM SN38, whereas doses greater than 50 nM were needed to induce the death of almost cells.

Next, we evaluate whether OSMI-4 could potentiate the cytotoxic effect of SN38. For this purpose, LeCo-2920vi tumoroïds were treated with 20 µM OSMI-4 alone or in combination with 10 or 25 nM SN38 for five days. At the end of the experiment, we assessed cell death through PDTOs diameter measurement (Fig. 6C, D) and caspase 3/7 activity (Fig. 6E). Despite a significant reduction in PDTOS diameters compare to control, OSMI-4 did not potentiate the cytotoxic effect of SN38 when used at a concentration of 10 nM. However, we observed a clear synthetic lethality when OSMI-4 was used in conjunction with a 25 nM SN38 treatment, as evidenced by a significant decrease in PDTOs diameter and drop in caspase 3/7 activity in comparison with PDTOs treated with OSMI-4 or SN38 alone, thereby validating our hypothesis. Importantly, we did not observe such an enhancement of SN38 cytotoxicity by OSMI-4 in organoids generated from healthy colon tissue (Supplementary Fig. S12), suggesting that OSMI-4 would not increase the side effects of chemotherapy on surrounding healthy tissue, and that this combinatorial treatment could potentially be considered in vivo.

## Discussion

The main goal of our study was to better understand the molecular mechanisms governing the entry of colon cancer cells into senescence in response to chemotherapy, a phenomenon increasingly recognized as contributing to treatment resistance and disease relapse [3]. While significant attention has been devoted to understanding the metabolism of cancer cells, the metabolic changes occurring during senescence and their consequences remained largely unexplored until recent times [18]. In this context, we focused on the HBP and *O*-GlcNAcylation processes which are now well-recognized hallmarks of cancers [24, 25] and that have already been shown to contribute to resistance to anticancer therapies by regulating different cell behaviors including drug efflux, epithelial-to-mesenchymal transition (EMT), apoptosis, autophagy and cell stemness [30].

Our findings revealed that entry into p53-dependent TIS of two colon cancer cell lines in response to two different chemotherapeutic agents was accompanied with a decrease in overall *O*-GlcNAcylation levels associated with reduced expression of GFAT and OGT (Fig. 1, Supplementary Fig. S3 and S4). Notably, a similar decrease in GFAT2 expression has been evidenced during HRAS^G12V^-mediated senescence of non-cancerous human bronchial epithelial cells (HBEC) [31] and we also observed this reduction in GFAT2 and *O*-GlcNAcylation levels during replicative senescence of normal human dermal fibroblasts (NHDFs) (datas not shown). This suggests that reprogramming of the HBP and *O*-GlcNAcylation is a general feature of senescence regardless of inducer and cell type.

Interestingly, the decrease in HBP activity observed during senescence coincides with an increase in glutaminolysis, indicating a diversion of glutamine away from the HBP towards glutaminolysis. Indeed, several studies have shown that several types of senescent cells, including replicative senescence, oncogene-induced senescence, and TIS cells, exhibit an enhanced utilization of glutamine as a mitochondrial fuel, facilitated by the upregulation of glutaminase 1, a key enzyme in the glutaminolysis pathway and that targeting this pathway could serve as a senolytic strategy [8, 15, 17, 32].

Additionally, we noted a decrease in OGA expression in senescent cancer cells **(**Fig. 1, Supplementary Fig. S3 and S4**)**. In a previous work, we showed that artificially modulating *O*-GlcNAcylation levels in colon cancer cells, led to compensatory adjustments in OGT and OGA in an attempt to restore basal *O*-GlcNAcylation levels [33]. This is likely the case in senescent cells as well.

Next, we delved into the significance of reprogramming the HPB and *O*-GlcNAcylation processes in establishing the senescence phenotype. Our results revealed that inducing a decrease in *O*-GlcNAcylation levels, either through GFAT or OGT inhibition, was not sufficient to induce entry into a senescent state in the absence of chemotherapy (Supplementary Fig. S6 and S7). Our results contrast with those reported by Taparra et al., who observed an increase in SA-β-galactosidase activity in H358, A549, and H460 lung cancer cells following pharmacological inhibition of GFAT or OGT, both in vitro and in xenograft experiments [31]. However, it’s important to note that in their study, they used DON and TT04, which are nonspecific inhibitors of GFAT and OGT, respectively, potentially accounting for this discrepancy.

We then investigated the consequences of OGA downregulation on the entry into TIS of colon cancer cells, hypothesizing that it could potentially delay or prevent the establishment of the senescence phenotype in response to low-dose chemotherapy. Surprisingly, our data clearly indicate that preventing the *O*-GlcNAcylation decrease normally occurring upon low-dose chemotherapy does not prevent cells from entering TIS (Fig. 2 and Supplementary Fig. S8). This suggests that the decrease in overall *O*-GlcNAcylation levels may have a minor role in TIS entry, although further investigations are needed to confirm this conclusion. Additionally, UDP-GlcNAc, the substrate of OGT, is also utilized by other GlcNAc transferases involved in complex glycosylation processes. Reduced GFAT expression and consequent UDP-GlcNAc levels likely impact these processes, potentially influencing the regulation of entry of colon cancer cells into TIS. In line with this hypothesis, Gitenay et al. demonstrated that supplementing the culture medium of immortalized human epithelial cells with N-Acetylglucosamine (GlcNAc) (which can serve as a substrate for the N-Acetylglucosamine kinase (NAGK) to directly regenerate UDP-GlcNAc through the GlcNAc salvage pathway) prevented their entry into OIS [34]. This result thus suggests a pivotal role of the HBP in the establishment of the senescence phenotype that warrants further exploration.

Lastly, we investigated the consequences of GFAT or OGT inhibition, hypothesizing this time, that it could potentiate the establishment of the senescence phenotype in response to low-dose chemotherapy. In line with this hypothesis, an increase in the percentage of OIS had been evidenced in HBEC cells and immortalized human epithelial cells, upon GFAT inhibition with DON or azaserine [31, 34]. In the same way, an increase in the percentage of radio-induced senescent breast cancer cells had also been observed upon the silencing of OGT [35]. On the contrary, we demonstrated that silencing GFAT or OGT, in conjunction with low-dose SN38 or etoposide treatment, induced a switch of HCT116 cells from senescence to apoptosis, going against our initial hypothesis (Figs. 3, 4 and Supplementary Fig. S9, S10). We also observed a dose dependent senescence-to-apoptosis switch in HCT116 and LS174T cells co-treated with the highly specific inhibitor of OGT, OSMI-4, and a low dose of SN38, thus validating the previous results (Fig. 5 and Supplementary Fig. S11).

Based on these results, we formulated the hypothesis that targeting *O*-GlcNAcylation in combination with chemotherapy could represent a promising synthetic lethality approach for CRC treatment, eliminating the risk of senescence escape and cancer relapse. To test this hypothesis, we evaluated whether OSMI-4 could enhance the cytotoxic effect of SN38 in a PDTO model, which has been demonstrated to be a valuable tool for predicting the clinical response to anticancer treatment. We observed a significant increase in PDTO cell death when OSMI-4 was used in combination with SN38, thus confirming our hypothesis (Fig. 6). This synthetic lethality between *O*-GlcNAcylation inhibition and conventional therapies has already been evidenced in other models in vitro and ex vivo [36–38], but to our knowledge, this is the first time it has been associated with the redirection of the therapeutic response from senescence to apoptosis.

We correlated this senescence-to-apoptosis switch to an enhancement of DNA damage in cells co-treated with *O*-GlcNAcylation inhibitors and low-dose chemotherapy in comparison with cells treated with chemotherapy alone. These results are in perfect accordance with recent literature suggesting that senescence induction in response to chemotherapy occurs within a specific range of DNA damage [28]. In line of this, 24 h after a 6 Gy irradiation of MCF7 breast cancer cells, Efimova et al. demonstrated that pharmacological inhibition of GFAT or OGT or knock-down of OGT was correlated with increased number of persisting Ionizing Radiation Inducing Foci (IRIF) compared to control cells thus suggesting that *O*-GlcNAcylation inhibition delay DNA repair [35, 39]. As mentioned earlier, in their experimental conditions, the authors observed an increase in the percentage of radio-induced senescent cells when OGT was silenced, suggesting that they were in a window of damage promoting senescence rather than apoptosis. However, all these results strongly suggest a role of *O*-GlcNAcylation in regulating the DNA damage response (DDR). Consistent with this hypothesis, in a study comparing the phosphoproteome of OGT knockout mouse embryonic fibroblasts (*vs* wild-type), several DDR pathway proteins (ATM, CHK1, 53BP1, and MDC1) were identified as hyperphosphorylated [40]. It has been shown that several proteins in this pathway are modified by *O*-GlcNAcylation: *O*-GlcNAcylation of ATM modulates its activation kinetics [41], while *O*-GlcNAcylation of H2AX and MDC1 decreases their phosphorylation, thereby preventing the spread of γH2AX foci around DNA breaks and aiding in their repair [42].

In conclusion, even though further investigations are required to better understand the repercussion of the reprogramming of the HBP and *O*-GlcNAcylation processes in the establishment of the senescent phenotype, our results strongly support that combining *O*-GlcNAcylation inhibition to conventional chemotherapies, to induce a senescence-apoptosis shift of cancer cells, could offer a promising strategy for MSI/dMMR/p53 unmutated CRC treatment potentially averting senescence escape and relapse. Furthermore, this approach may increase treatment specificity, as colorectal cancer cells exhibit higher *O*-GlcNAcylation levels compared to normal tissues.

## Material and methods

### Cell culture

HCT116 cells obtained from ATCC were cultured in McCoy’s 5 A (modified) medium supplemented with Glutamax (Thermofischer Scientific), 10% fetal calf serum, and 1% ZellShieldTM (Biovalley). Cells were cultured at 37 °C in a 5% CO2 atmosphere with saturated humidity.

### Chemicals

The OGT inhibitor, OSMI-4 was purchased from Medchem Express (Cat. No.: HY-114361), SN38 from Biotechne (Cat. No.: 2684/10) and Etoposide from Sigma-Aldrich (Cat. No.: E1383). They were all dissolved in DMSO.

### Small interfering RNA

HCT116 cells were reverse-transfected using Lipofectamine RNAiMax (Invitrogen), following the manufacturer’s protocol. They were treated with either 5 nM small interfering RNA (siRNA) targeting OGT (siGENOME human OGT siRNA D-019111-01, Dharmacon), OGA (siGENOME human MGEA5 D-012805-01, Dharmacon), GFAT1 (siGENOME human GFPT1 siRNA M-008833-01, Dharmacon), GFAT2 (siGENOME human GFPT2 M-010390-01, Dharmacon), or a scrambled control sequence (siCTRL; siGENOME RISC free control siRNA, Dharmacon), as previously described [21]. The cells were harvested for RNA/protein extraction either 72 or 96 h post-transfection, depending on the experiment.

### Quantitative RT-PCR

RNA extraction was carried out using the Nucleospin® RNA mini spin kit (Macherey-Nagel) following the manufacturer’s protocol. Subsequently, 1 µg of total RNA underwent reverse transcription using random primers and MultiScribeTM reverse transcriptase (Applied Biosystems). Real-time PCR analysis was conducted with Power SYBR Green (Applied Biosystems) on a QuantStudio 3 fluorescence temperature cycler (ThermoFisher Scientific), as per the manufacturer’s guidelines. Data were normalized using Expressed Alu Repeats (EAR) amplification [43]. The primer sequences employed for the RT-qPCR analyses are detailed in Supplementary Table S1.

### Proteins extraction, Western blotting and antibodies

For total protein extraction, cells were lysed using RIPA buffer (10 mM Tris [pH 7.4], 150 mM NaCl, 1 mM EDTA, 1% Triton X-100, 0.5% sodium deoxycholate, 0.1% SDS, and protease inhibitors added at the time of preparation). Protein concentration was quantified using the Micro BCA Protein Assay Kit (Thermofisher Scientific). Equal amounts of proteins were then separated by SDS-PAGE and transferred onto nitrocellulose membranes (GE Healthcare). Following a 1 h blocking step in PBSM (PBS with 5% defatted powdered milk), the membranes were incubated overnight at 4 °C with specific primary antibodies in PBSTM (PBSM with 0.1% Tween 20) and washed three times with PBSN (PBS with 0.1% NP-40). Subsequently, the membranes were incubated for 1 h at room temperature with secondary antibodies coupled to peroxidase in PBSM, washed twice in PBSN and once in PBS, and then visualized via chemiluminescence. Mouse monoclonal anti-*O*-GlcNAc (RL2) was purchased from Life Technologies (MA1072), Rabbit polyclonal anti-OGT (DM17) from Sigma-Aldrich (#O6264), mouse monoclonal anti-GAPDH (sc-32223) and anti-chicken IgY-HRP (sc-2428) from Santa Cruz Biotechnology, rabbit polyclonal anti-p21 (#2947), anti-EZH2 (#5246) and cyclin D1 (#2922) from cell signaling, rabbit polyclonal anti-γH2AX from Novus Biologicals (NB 100-384), mouse monoclonal anti-caspase 7 from Enzo life science (ADI-AAM 127 E), rabbit monoclonal anti-PARP1 from Abcam (ab32138), anti-mouse IgG-HRP from GE Healthcare (NA931V) and donkey anti-rabbit IgG-HRP from Millipore (AP182P). Antibodies for OGA (345) were gracious gifts from the laboratory of Gerald Hart (Johns Hopkins University, Baltimore, Maryland, USA).

### SA-beta-galactosidase (SA-β-Gal) activity assays

#### Colorimetric assay

Cells were washed in PBS, fixed for 10 minutes in 4% paraformaldehyde at room temperature, washed three times in PBS, and then incubated for 16 h at 37 °C (without CO2) in a SA-β-Gal stain solution containing 1 mg/ml 5-bromo-4-chloro-3-indolyl-β-D-galactoside (X-Gal, Euromedex EU0012-D), 40 mM sodium mono-diphosphate (pH 6), 5 mM potassium ferrocyanide, 5 mM potassium ferricyanide, 150 mM NaCl, and 2 mM MgCl2. Senescent cells exhibit a blue staining.

#### Fluorometric assay

Cells were trypsinized, resuspended in PBS, and manually counted. For each experimental condition, 2000 cells were transferred in triplicate to a 1.5 ml tube. SA-β-gal activity was assessed using the cellular senescence activity assay kit (Enzo, ENZ-KIT 129) following the instructions provided in the product manual.

### Cell viability assay

Cell viability was assessed using the MTT assay with 40,000 HCT116 cells plated in a 6-well plate. A stock solution of 5 mg/mL MTT (3-(4,5-dimethylthiazol-2-yl)-2,5-diphenyltetrazolium bromide) was prepared in PBS and then diluted 1:10 with culture medium. Subsequently, 500 µL of this diluted MTT solution was added to each well, followed by a 2 h incubation at 37 °C. After incubation, the supernatant was removed, and a lysis solution of isopropanol/HCl (23:1) was added, with further incubation for 30 min at 37 °C. Following this, 100 µL of the lysate was transferred to a 96-well plate in triplicate, and the absorbance was measured at 540 and 620 nm. The intensity of the product color, measured as the difference between absorbance at 540 nm and 620 nm, is directly proportional to the number of living cells in the culture.

### PDTOs obtention and culture

Patient-Derived Tumor Organoïds (PDTOs) originating from a sigmoid colon tumor (referred to as LeCo-2920vi) were generated by the OrgaRES Platform using a tumor sample obtained from a female patient aged 76 undergoing surgery in the Department of Digestive Surgery and Transplantation of the Lille University Hospital, as described in [44]. The resected tumor was histologically diagnosed as a well-differentiated pT3N0, Grade IIA sigmoid cancer. Importantly, the patient had not received any neoadjuvant chemotherapy.

For subculturing, PDTOs were harvested and dissociated into single cells by using trypsin-EDTA 0.25%, then resuspended in Matrigel (Corning #356231) and seeded in 40 μL domes within wells of a 24-well plate. Once the Matrigel solidified, the domes were covered with complete colon tumor organoid medium (Advanced DMEM/F12 (ADF, Invitrogen #11320-082) supplemented with Glutamax [1×, Invitrogen #35050-061], HEPES buffer [1×, Sigma–Aldrich #83264-100ML-F], Penicillin/Streptomycin [1X, Invitrogen #5070-063], B-27 Supplement Minus Vitamin A (Invitrogen #12587-010), N2 supplement [1 x, Invitrogen #17502-048], N-acetyl-L-cysteine [1 mM, Sigma–Aldrich], mouse recombinant epidermal growth factor [EGF, 50 ng/ml, Invitrogen #PMG8041], mouse recombinant Noggin [100 ng/ml, Preprotech #250-38], Rho Kinase inhibitor Y27632 [10 µM, BD Biosciences #562822] ALK-5 and Smad signaling inhibitor A83-01 [0.5 μM, Tocris #2939], Nicotinamide [10 mM, Sigma–Aldrich #N0636], Gastrin [10 nM, Sigma–Aldrich #G9145] and p38 MAPK inhibitor SB202190 [10 µM, Sigma #S7067]. Complete medium was refreshed every 2 days, and PDTOs were passaged through mechanical disruption every 2 weeks.

### Cell viability and caspase 3/7 assays in PDTOs

PDTOs were harvested and dissociated into single cells by using trypsin-EDTA 0.25%. 2000 single cells per 20 µl Matrigel dome were plated in 96 well opaque plates (Greiner bio one, #655090) and allowed to grow for fifteen days before the different treatments. For the cell viability assay, PDTOs were treated with increasing concentrations of SN38 ranging from 5 to 100 nM for 72 h. The viability of PDTOs was then evaluated using the CellTiter-Glo® 3D Cell Viability Assay kit (Promega), following the instructions provided by the manufacturer. For the caspase 3/7 activity, PDTOs were treated with 20 µM OSMI-4 alone or in combination with 10 or 25 nM SN38 for 72 h. Caspase 3/7 activity was then assessed using the Caspase-Glo® 3/7 Assay kit from Promega, following the manufacturer’s instructions.

### Statistical analysis

The in vitro experiments were performed at least three times independently (*n* = 3). The PDTOs experiments were repeated at least twice (*n* = 2). Results are presented as individual values and mean +/- SEM or SD depending on the experiments. One-way ANOVA was used to determine statistical significance among multiple groups. Two-tailed unpaired Student’s *t*-test was used for comparisons between two groups. Statistical analyses were performed with the GraphPad prism software version 10.2.3. Statistical significance was indicated as *P < 0.05, **P < 0.01, ***P < 0.001, ****P < 0.0001.

### Supplementary files

This article contains supplementary Figures and Material including the full and uncropped Western Blots presented in the study.

### Methods statement

All methods were performed in accordance with the relevant guidelines and regulations.

## Supplementary information

Loison et al. supplementary files

Loison et al. 2024 non cropped blots
