# Supplementary material for: O-GlcNAcylation inhibition redirects the response of colon cancer cells to chemotherapy from senescence to apoptosis
Source: Cell Death Dis. 2024 Oct 19;15(10):762. doi: 10.1038/s41419-024-07131-5 (PMC11490504; doi:10.1038/s41419-024-07131-5)
Supplement: Supplementary file 1 — Loison et al. supplementary files [file 41419_2024_7131_MOESM1_ESM.pdf]

**Supplementary Figure S1: Low-dose SN38-treated HCT116 cells are sensitive to a senolytic treatment.**

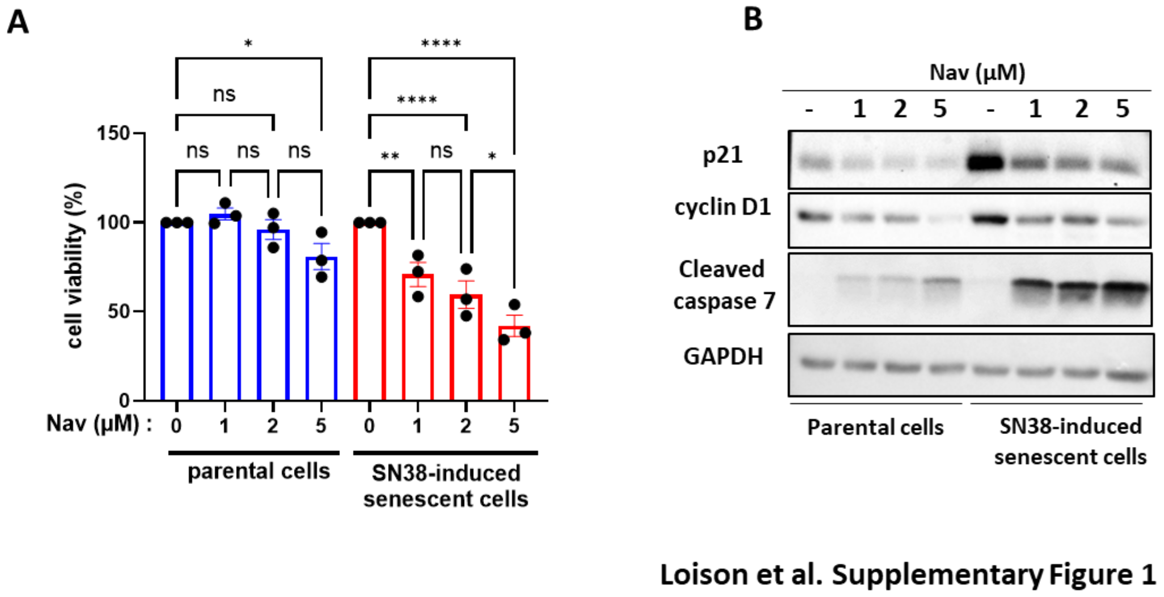


Proliferating parental HCT116 cells and SN38-inducent senescent cells were treated with increasing concentration of navitoclax (Nav), a well characterized senolytic, for 24 hours **(A)** Cell viability was assessed through the MTT assay. For this, cells were incubated with an MTT solution to achieve a final concentration of 0.5 mg/ml in the culture medium and incubated at 37°C for 2 hours. Following this, the cells were lysed using a solution of isopropanol/HCl 1N, at a 23:1 ratio, and further incubated at 37°C for 30 minutes. For each condition, 100 µl of the lysate were deposited in a 96-well plate, and the optical density was measured at 540 nm and 620 nm. Cell viability was calculated by subtracting the OD measured at 620 nm from that at 540 nm for each sample, with the control (non-treated cells) set to 100%. Results represent the individual values and mean +/- SEM of three independent experiments (n=3) (ns: non-

significant, *P<0.05,**P<0.01,****P<0.0001, ordinary one-way ANOVA with Fisher's LSD multiple comparison tests). **(B)** Western blot analysis was performed to assess the expression of the senescence markers p21 and cyclin D1 and the apoptosis marker cleaved-caspase 7. GAPDH was used as a loading control. Results are representative of three independent experiments (n=3).

**Supplementary Figure S2: SN38-induced senescence of HCT116 cells rely on a functional p53 pathway.**

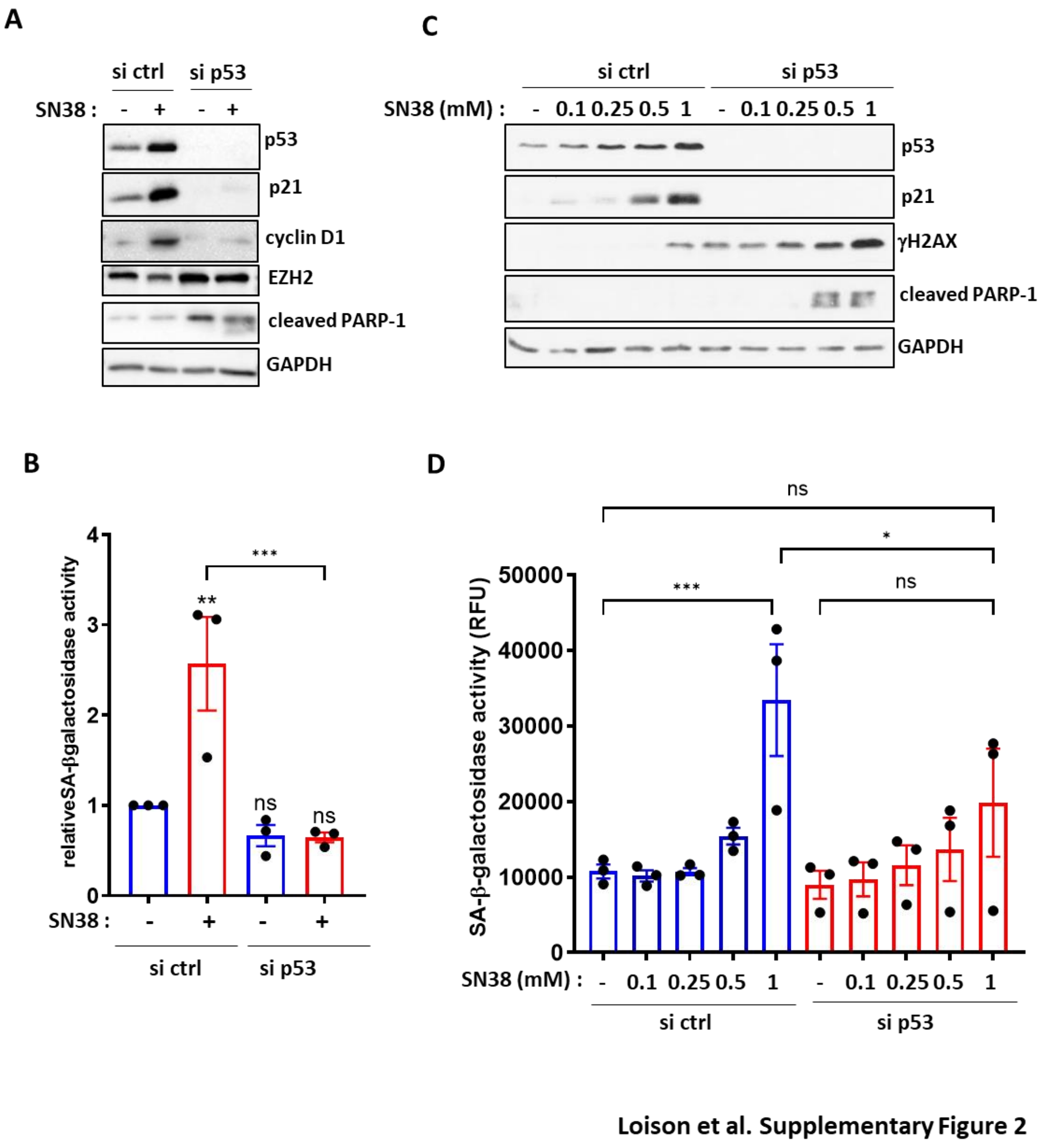


**(A-B)** HCT116 cells were transfected with a siRNA targeting TP53 (si p53, siGENOME human TP53 siRNA M-003329-03 Dharmacon) or a non-target control siRNA (siCTRL). 24 hours later, the cells were treated with 1 nM SN38 for 72 hours. **(A)** Western blot analysis was performed to assess the expression of the three senescence markers p21, cyclin D1 and EZH2,

the DNA damage sensor γH2AX and the apoptosis markers cleaved-caspase 7 and cleaved-PARP1. The efficiency of siRNA was also confirmed by evaluating p53 loss of expression. GAPDH was used as a loading control. Results shown are representative of three independent experiments (n=3). **(B)** Quantitative fluorimetric determination of the SA-β-Galactosidase activity in the different experimental conditions. Results represent the individual values and mean +/- SEM of three independent experiments (n=3) (ns: non-significant, **P<0.01, ***P<0.001 ordinary one-way ANOVA with Fisher's LSD multiple comparison tests). **(C-D)** HCT116 cells were transfected with a siRNA targeting TP53 (si p53, siGENOME human TP53 siRNA M-003329-03 Dharmacon) or a non-target control siRNA (siCTRL). 24 hours later, the cells were treated with increasing concentration of SN38 ranging from 0.1 to 1 nM for 72 hours. **(C)** Western blot analysis was performed to assess the expression of p53, p21, γH2AX and cleaved-PARP1. GAPDH was used as a loading control. Results shown are representative of two independent experiments (n=2). **(B)** Quantitative fluorimetric determination of the SA-β-Galactosidase activity in the different experimental conditions. Results represent the individual values and mean +/- SEM of two independent experiments (n=2) (ns: non-significant, **P<0.01, ***P<0.001 ordinary one-way ANOVA with Fisher's LSD multiple comparison tests)

**Supplementary Figure S3: etoposide-induced senescence of HCT116 cells is accompanied by a decrease in GFAT2, OGT, OGA, and *O*-GlcNAcylation levels.**

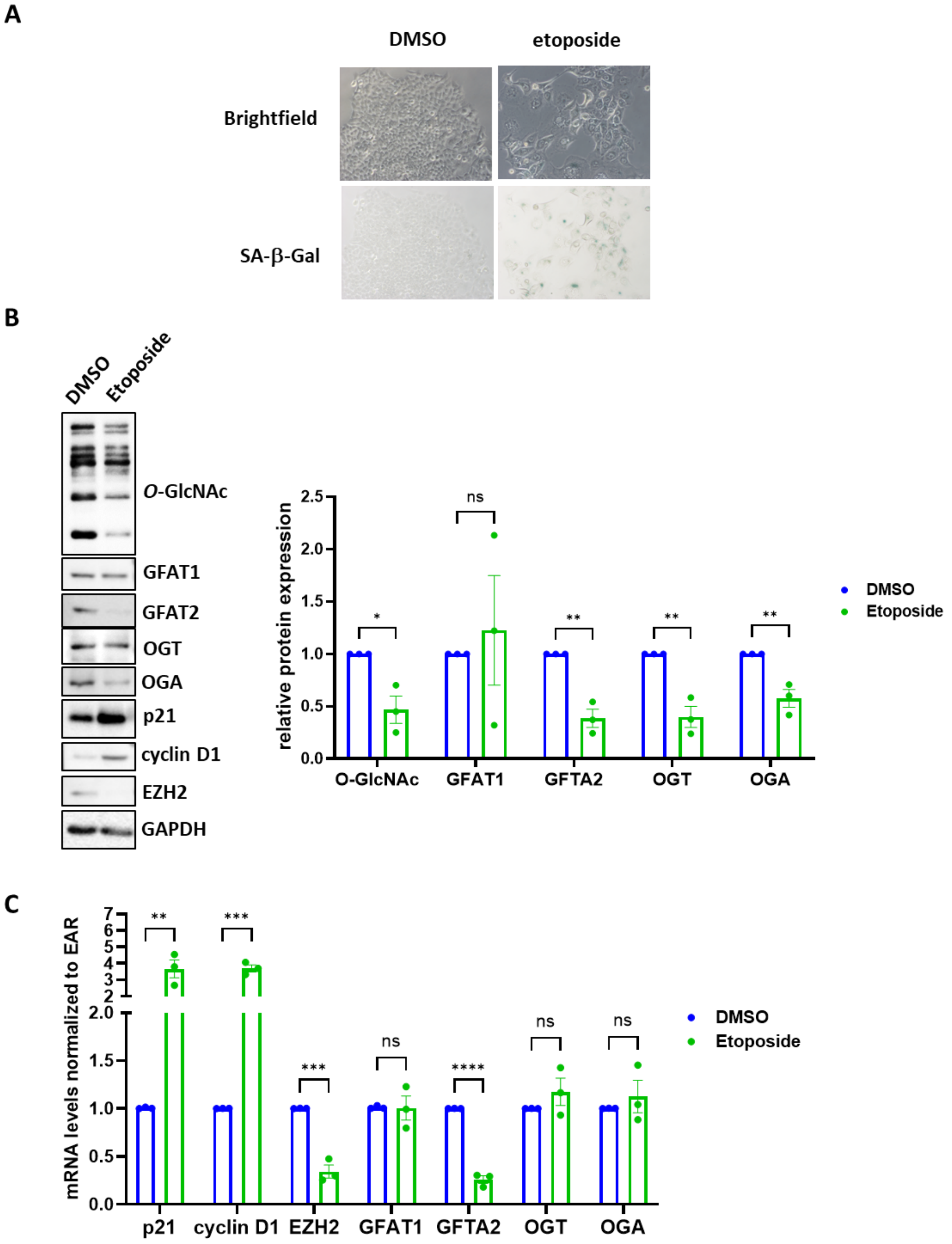


Loison et al. Supplementary Figure 3

HCT116 cells were treated with 2 µM etoposide for 96 hours or with DMSO as a negative control. **(A)** Top: Representative images of the microscopic analysis of cell morphology showing an increase in the size of senescent cells. Pictures were taken at the same magnification (x200) Bottom: SA-β-Galactosidase activity assay demonstrating blue staining of senescent cells. **(B)** Left: Western blot analysis of *O*-GlcNAcylation levels, OGT, OGA, and both isoforms of GFAT (GFAT1 and GFAT2), as well as the expression of p21, cyclin D1, and EZH2, three senescence markers. Right: Quantification of relative protein expression from three independent experiments (optical density measurement relative to GAPDH) (n=3) (individual values and mean +/- SEM, ns: non-significant, *P<0.05, **P<0.01, multiple unpaired t-tests). **(E)**: qRT-PCR analysis of p21, cyclin D1, GFAT1, EZH2, GFAT2, OGT, and OGA transcript expression. Results represent the individual values and mean +/- SEM of three independent experiments (n=3) (ns: non-significant***P<0.001, ****P<0.0001, multiple unpaired t-tests).

**Supplementary Figure S4: SN38 or etoposide-induced senescence of LS174T cells is accompanied by a decrease in GFAT1, OGT, OGA, and *O*-GlcNAcylation levels.**

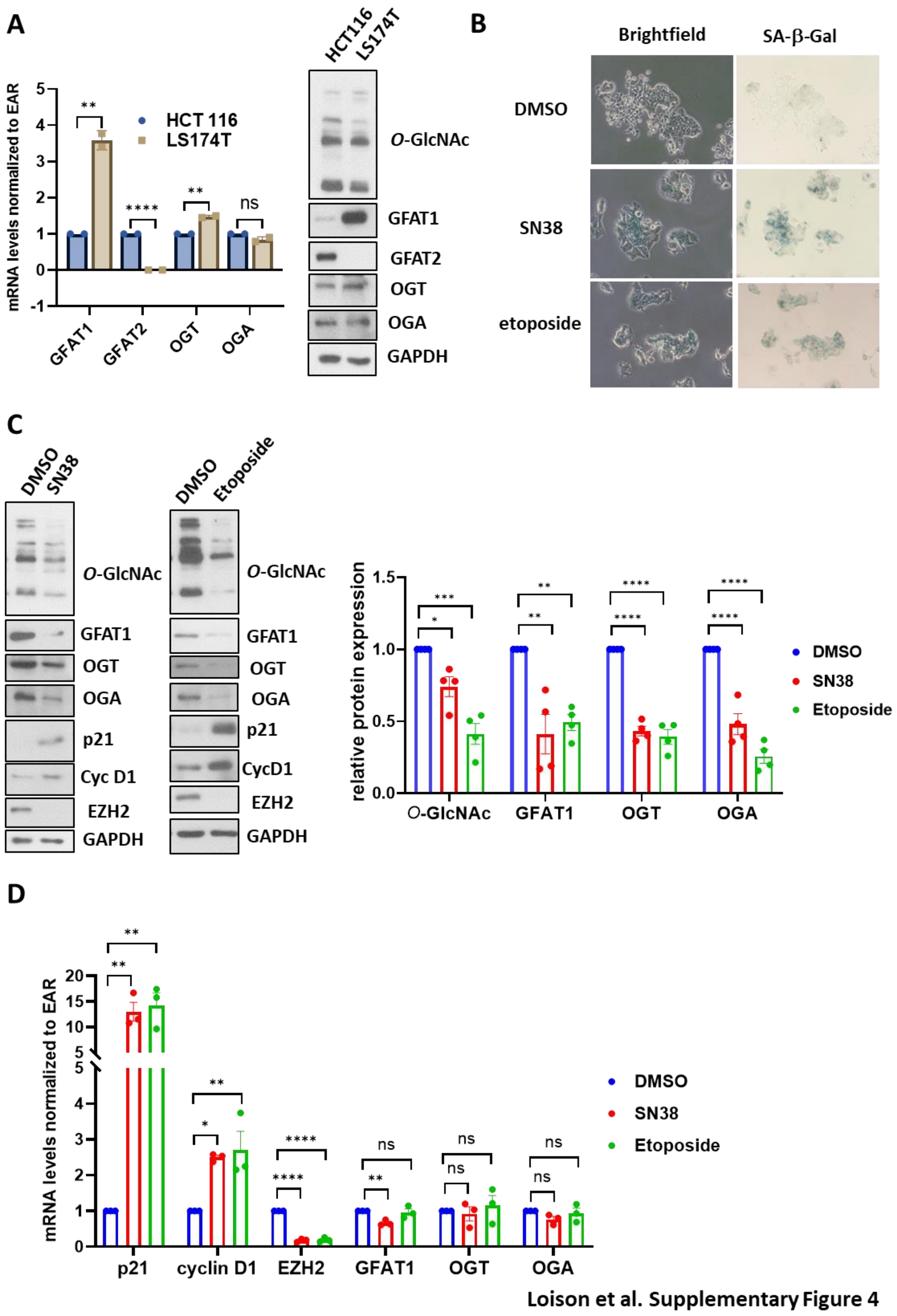

LS174T cells (obtained from Pr Olivier Coqueret's lab) were maintained in EMEM (Lonza) supplemented with 10% fetal calf serum and 1 % ZellShieldTM (Biovalley). Cells were cultured at 37°C in water-saturated 5% $CO_2$ atmosphere. **(A)** Comparative analysis of the expression of GFAT1, GFAT2, OGT, OGA and *O*-GlcNAcylation levels at the mRNA (left) and protein level (right) between HCT116 and LS174T cells. The qRT-PCR results represent the individual values and mean +/- SD of two independent experiments (n=2) (ns: non-significant, **P<0.01, ***P<0.001, multiple unpaired t-tests). Western Blot analyses are representative of the two independent experiments **(B-D)** Cells were treated with either 5 nM SN38 or 1 µM etoposide for 72 hours or with DMSO as a negative control. **(B)** Left: Representative images of the microscopic analysis of cell morphology showing an increase in the size of senescent cells. Pictures were taken at the same magnification (x200). Right: SA-β-Galactosidase activity assay demonstrating blue staining of senescent cells. **(C)** Left: Western blot analysis of *O*-GlcNAcylation levels, OGT, OGA, and GFAT1 as well as the expression of p21, cyclin D1, and EZH2, three senescence markers. Right: Quantification of relative protein expression from four independent experiments (optical density measurement relative to GAPDH) (n=4) (individual values and mean +/- SEM, ns: non-significant, *P<0.05, **P<0.01, ***P<0.001, ****P<0.0001, ordinary one-way ANOVA with Fisher's LSD multiple comparisons tests). **(D)**: qRT-PCR analysis of p21, cyclin D1, EZH2, GFAT1, OGT, and OGA transcript expression. Results represent the individual values and mean +/- SEM of three independent experiments

(n=3) (ns: non-significant, *P<0.05, **P<0.01, ****P<0.0001, ordinary one-way ANOVA with Fisher's LSD multiple comparisons tests).

**Supplementary Figure S5: SN38-induced senescence of HT29 and Caco2 cells is not accompanied by a reprogramming of the HBP pathway or *O*-GlcNAcylation processes.**

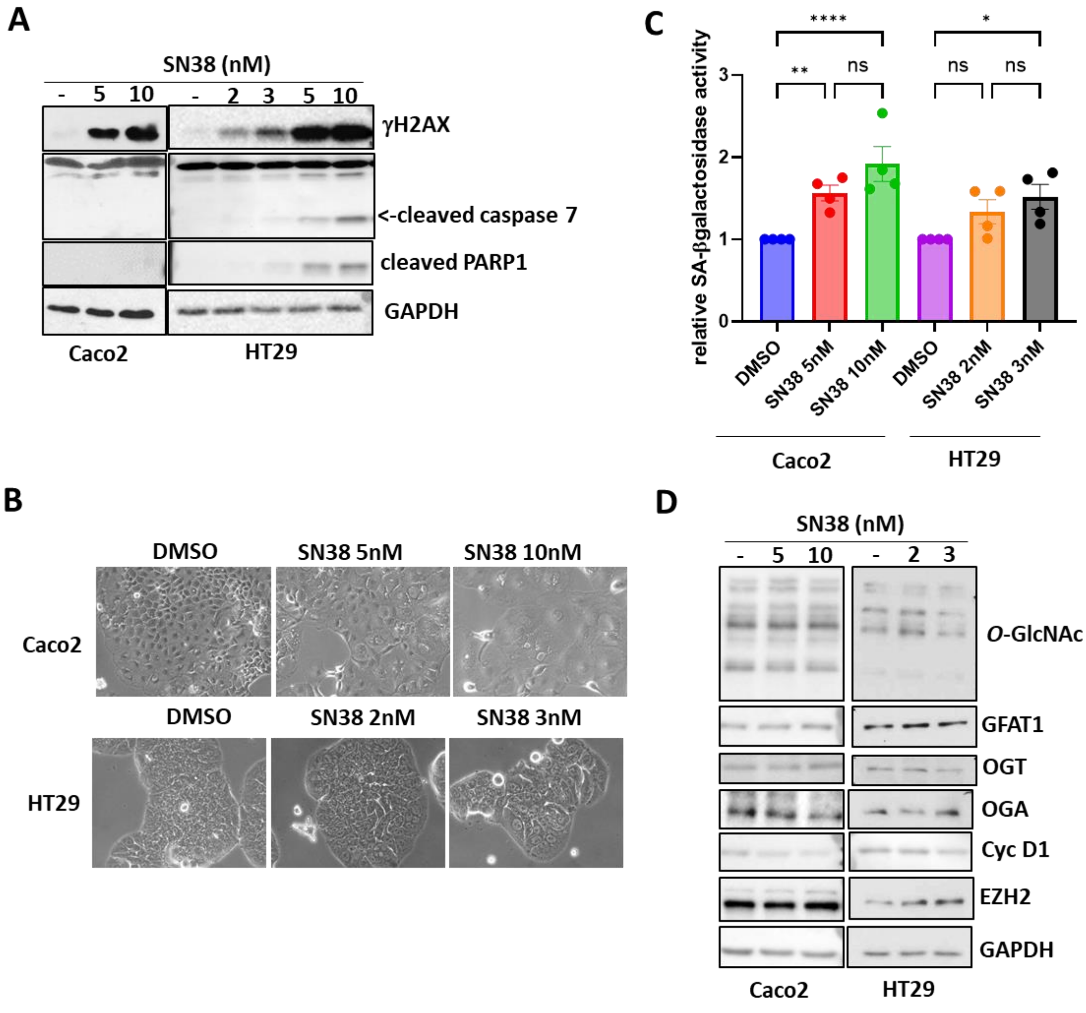


**Loison et al. Supplementary Figure 5**

Caco2 and HT29 cells were purchased from ATCC. Caco2 were maintained in DMEM supplemented with with Glutamax (Thermofischer Scientific), 10% fetal calf serum and 1 % ZellShieldTM (Biovalley). HT29 cells were cultured in McCoy's 5A (modified) medium

supplemented with Glutamax (Thermofischer Scientific), 10% fetal calf serum, and 1% ZellShieldTM (Biovalley). The two cell lines were cultured at 37°C in a 5% CO2 atmosphere with saturated humidity. **(A)** Cellls were treated with increasing concentrations of SN38 for 72h (Caco2) or 96 hours (HT29). Cell apoptosis was investigated by Western Blot analyses of cleaved-caspase 7 and cleaved-PARP1. GAPDH was used as a loading control. Data shown are representative of two independent experiments. **(B)** Representative images of the microscopic analysis of cell morphology showing a moderate increase in the size of SN38-treated cells. Pictures were taken at the same magnification (x200). **(C)** Quantitative fluorimetric determination of the SA-β-Galactosidase activity showing a dose-dependent slight increase in response to SN38 treatment. Results represent the individual values and mean +/- SEM of four independent experiments (n=4) (ns: non-significant, *P <0.05, **P<0.01, ****P<0.0001, ordinary one-way ANOVA with Fisher's LSD multiple comparison tests). **(D)** Western blot analysis of *O*-GlcNAcylation levels, OGT, OGA, and GFAT1 as well as the expression of cyclin D1, and EZH2, two senescence markers showing no difference whatever the experimental condition. Results are representative of three independent experiments (n=3).

**Supplementary Figure S6: Knock-down of GAT1 and GFAT2 alone or in combination are not sufficient to induce senescence of HCT116 cells in the absence of chemotherapy.**

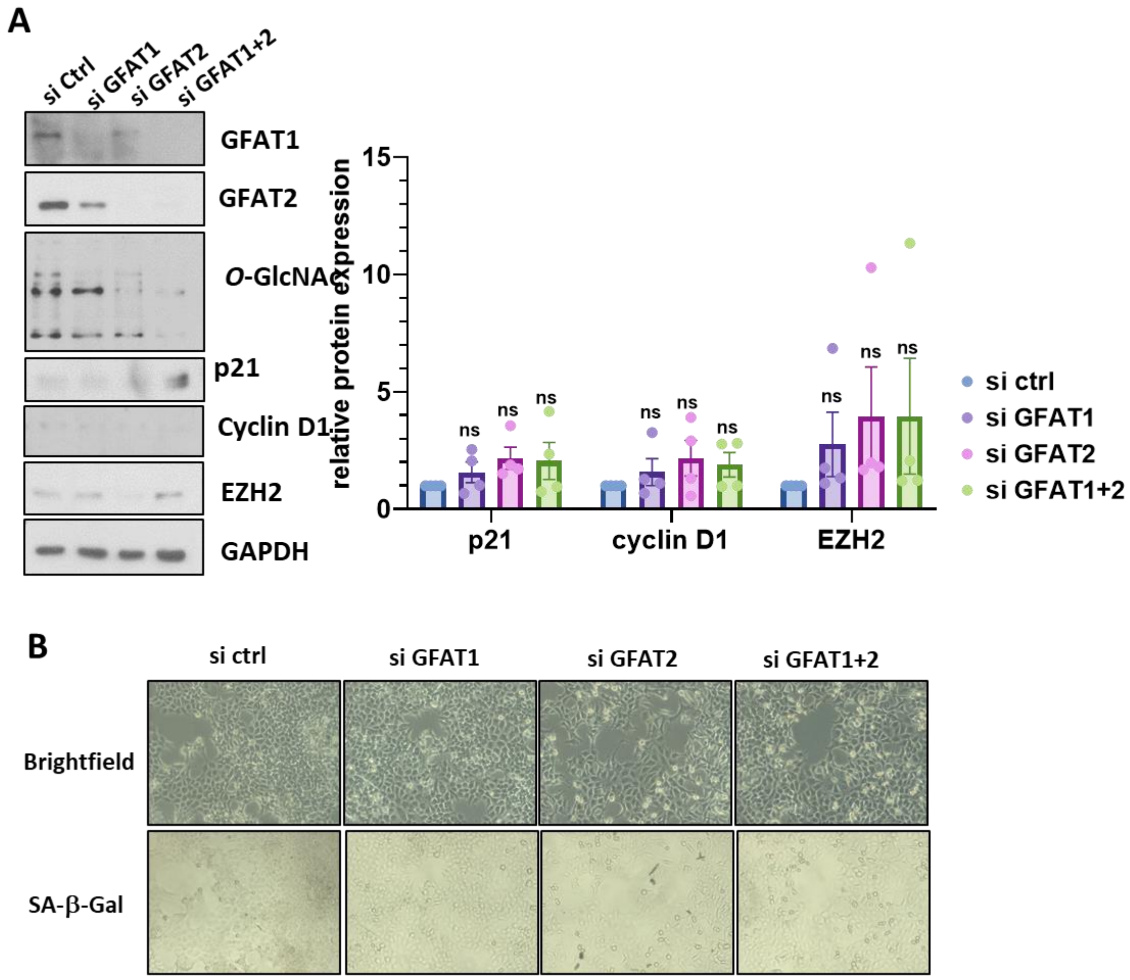


Loison et al. Supplementary Figure 6

HCT116 cells were transfected with siRNA targeting GFAT1 or GFAT2 alone (si GFAT1 or si GFAT2) or in combination (si GFAT1+2) or a non-target control siRNA (siCTRL) for 96 hours. **(A)** Left**:** Western blot analysis was performed to assess the expression of the three senescence markers p21, cyclin D1, and EZH2. The efficiency of siRNA was also confirmed by evaluating GFAT1, GFAT2 and *O*-GlcNAcylation levels. GAPDH was used as a loading control. Results

shown are representative of four independent experiments. Right: quantification of relative protein expression analyzed by Western Blot (optical density measurement relative to GAPDH) of P21, Cyclin D1 and EZH2 from four independent experiments (n=4) (individual values and mean +/- SEM, ns: non-significant, ordinary one-way ANOVA with Dunnet's multiple comparisons tests). **(B)** Top: Representative images of the microscopic analysis of cell morphology showing no difference in the size cells whatever the condition. Pictures were taken at the same magnification (x200). Bottom: SA-β-Galactosidase activity assay showing no blue staining characteristic of senescent cells.

**Supplementary Figure S7: Inhibiting the expression or the activity of OGT is not sufficient to induce senescence of HCT116 cells in the absence of chemotherapy.**

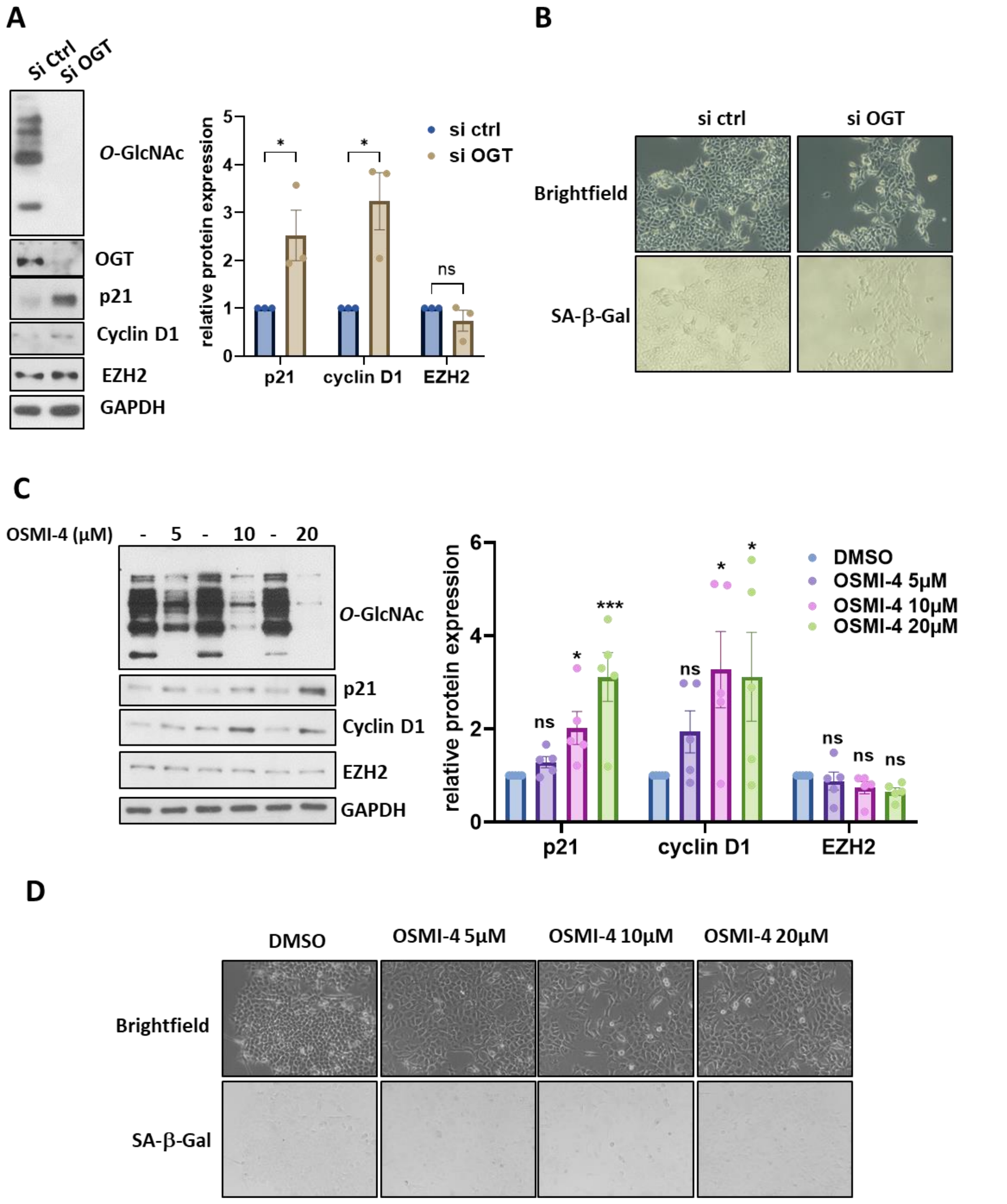


Loison et al. Supplementary Figure 7

**(A-B)**: HCT116 cells were transfected with siRNA targeting OGT (si OGT) or a non-target control siRNA (siCTRL) for 72 hours. Results are representative of three independent experiments (n=3) **(C-D)** HCT116 cells were treated with increasing concentrations of the OGT inhibitor OSMI-4 ranging from 5 to 20 µM for 72 hours. Results are representative of five independent experiments (n=5) **(A, C)** Left**:** Western blot analysis was performed to assess the expression of the three senescence markers p21, cyclin D1, and EZH2. Right: quantification of relative protein expression analyzed by Western Blot (optical density measurement relative to GAPDH) of P21, Cyclin D1 and EZH2 (individual values and mean +/- SEM, ns: non-significant, *P<0.05, ***P<0.001, multiple unpaired t-tests for si OGT experiments and ordinary one-way ANOVA with Dunnet's multiple comparisons tests for OSMI-4 experiments) **(A)** The efficiency of siRNA was also confirmed by evaluating OGT and *O*-GlcNAcylation levels. **(C)** The efficiency of the OSMI-4 treatment was ensured through the evaluation of the *O*-GlcNAcylation levels. GAPDH was used as a loading control. **(B, D)** Top: Representative images of the microscopic analysis of cell morphology showing no difference in the size cells whatever the condition. Pictures were taken at the same magnification (x200). Bottom: SA-β-Galactosidase activity assay showing no blue staining characteristic of senescent cells.

**Supplementary Figure S8: Preventing *O*-GlcNAcylation decrease through OGA silencing does not impact the entry of HCT116 cells into etoposide-induced senescence.**

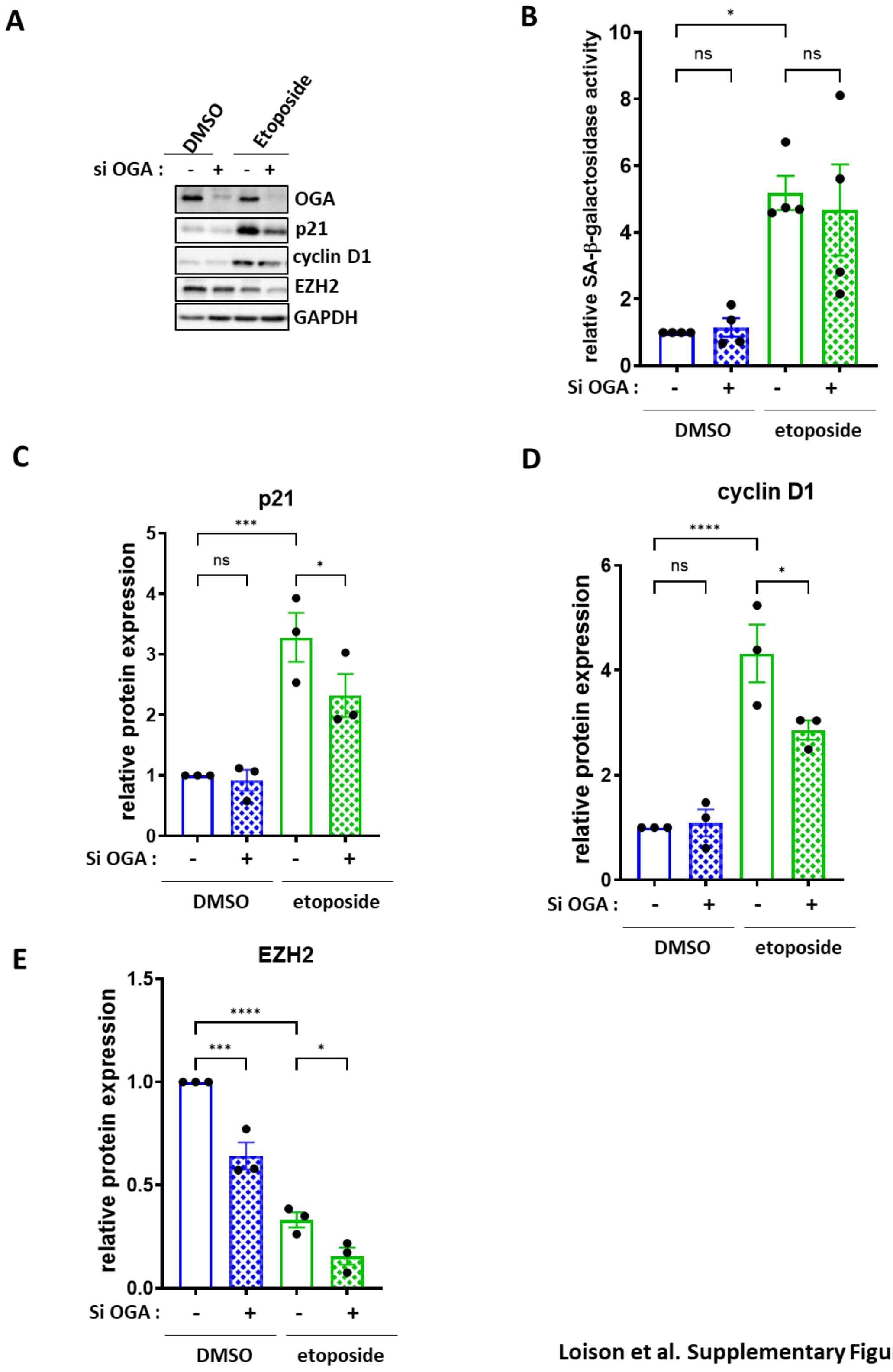


Loison et al. Supplementary Figure 8

HCT116 cells were transfected with a siRNA targeting OGA (si OGA) or a non-target control siRNA (siCTRL). 24 hours later, the cells were treated with 1 µM etoposide for 72 hours. **(A)** Western blot analysis was performed to assess the expression of the senescence markers p21 and cyclin D1. The efficiency of siRNA was also confirmed by evaluating OGA loss of expression. GAPDH was used as a loading control. Results shown are representative of three independent experiments. **(B)** Quantitative fluorimetric determination of the SA-β-Galactosidase activity in the different experimental conditions. RFU: Relative Florescence Unit. Results represent the individual values and mean +/- SEM of four independent experiments (n=4) (ns: non-significant, **P <0.01, ordinary one-way ANOVA with Fisher's LSD multiple comparison tests). **(C-E)**: quantification of relative protein expression analyzed by Western Blot (optical density measurement relative to GAPDH) of p21 **(C)**, cyclin D1 **(D)** and EZH2 **(E)** from three independent experiments (n=3) (individual values and mean +/- SEM, ns: non-significant, *P <0.05, ***P<0.001, ****P<0.0001, ordinary one-way ANOVA with Fisher's LSD multiple comparison tests).

**Supplementary Figure S9: Silencing GFAT1 and GFAT2 prevents the entry of HCT116 cells into etoposide-induced senescence and induces their apoptosis through enhancement of DNA damage.**

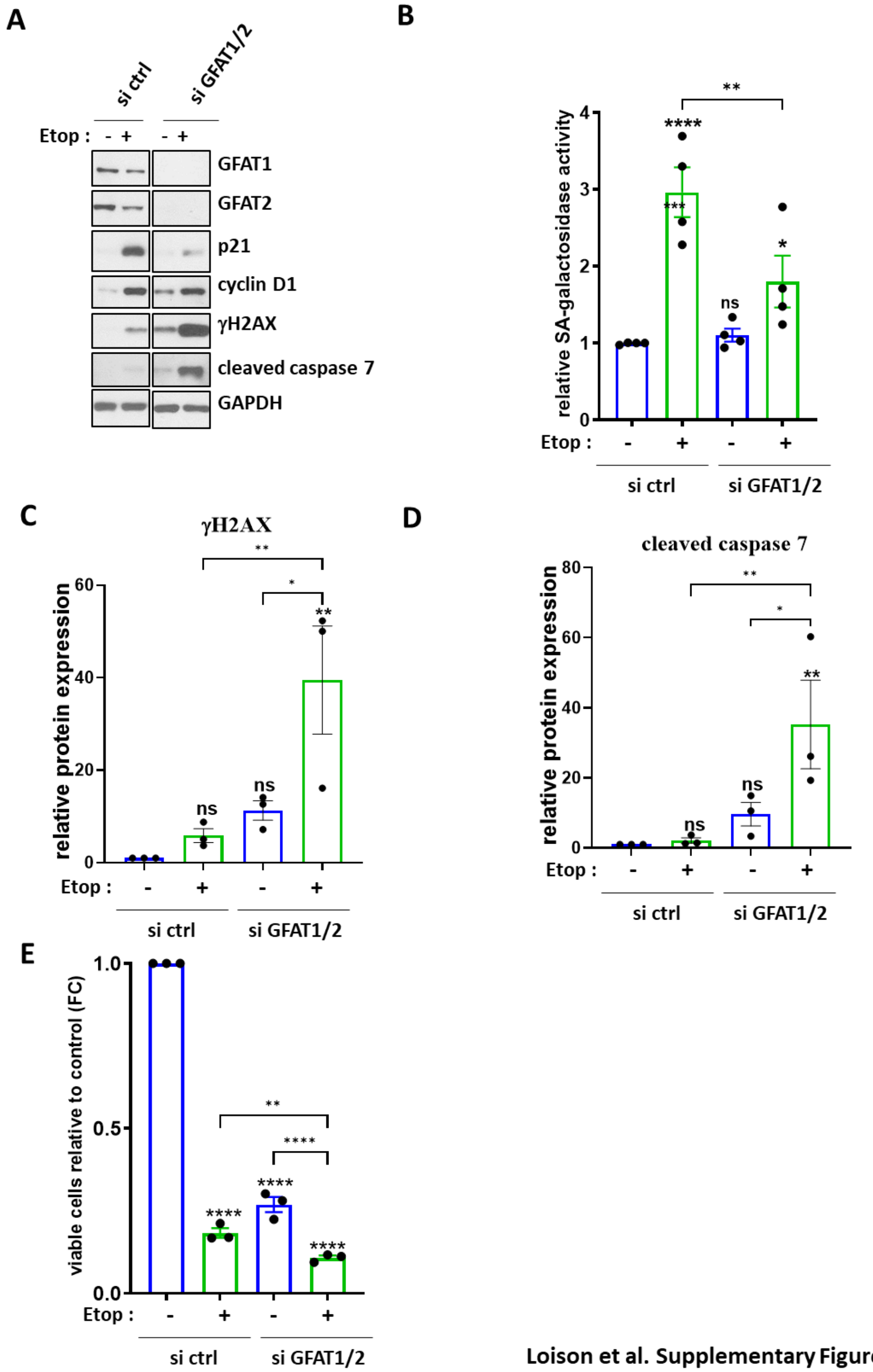


Loison et al. Supplementary Figure 9

HCT116 cells were transfected with a siRNA targeting GFAT1 and GFAT2 (si GFAT1/2) or a non-target control siRNA (siCTRL). 24 hours later, the cells were treated with 1 µM etoposide for 72 hours. **(A)** Western blot analysis was performed to assess the expression of the two senescence markers p21 and cyclin D1, the DNA damage sensor γH2AX and the apoptosis marker cleaved-caspase 7. The efficiency of siRNA was also confirmed by evaluating GFAT1 and GFAT2 loss of expression. GAPDH was used as a loading control. Results shown are representative of three independent experiments. **(B)** Quantitative fluorimetric determination of the SA-β-Galactosidase activity in the different experimental conditions. RFU: Relative Florescence Unit. Results represent the individual values and mean +/- SEM of four independent experiments (n=4) (ns: non-significant, *P<0.05, **P<0.01, ****P<0.0001 ordinary one-way ANOVA with Fisher's LSD multiple comparison tests). **(C-D)**: quantification of relative protein expression analyzed by Western Blot (optical density measurement relative to GAPDH) of γH2AX **(C)** and cleaved-caspase 7 **(D)** from three independent experiments (n=3) (individual values and mean +/- SEM, ns: non-significant, *P <0.05, **P<0.01, ordinary one-way ANOVA with Fisher's LSD multiple comparison tests). **(E)** Quantification of the ratio of viable cells through MTT assay from three independent experiments (n=3) (individual values and mean +/- SEM, **P<0.01, ****P<0.0001, ordinary one-way ANOVA with Fisher's LSD multiple comparison tests).

**Supplementary Figure S10: Silencing OGT prevents the entry of HCT116 cells into etoposide-induced senescence and induces their apoptosis through enhancement of DNA damage.**

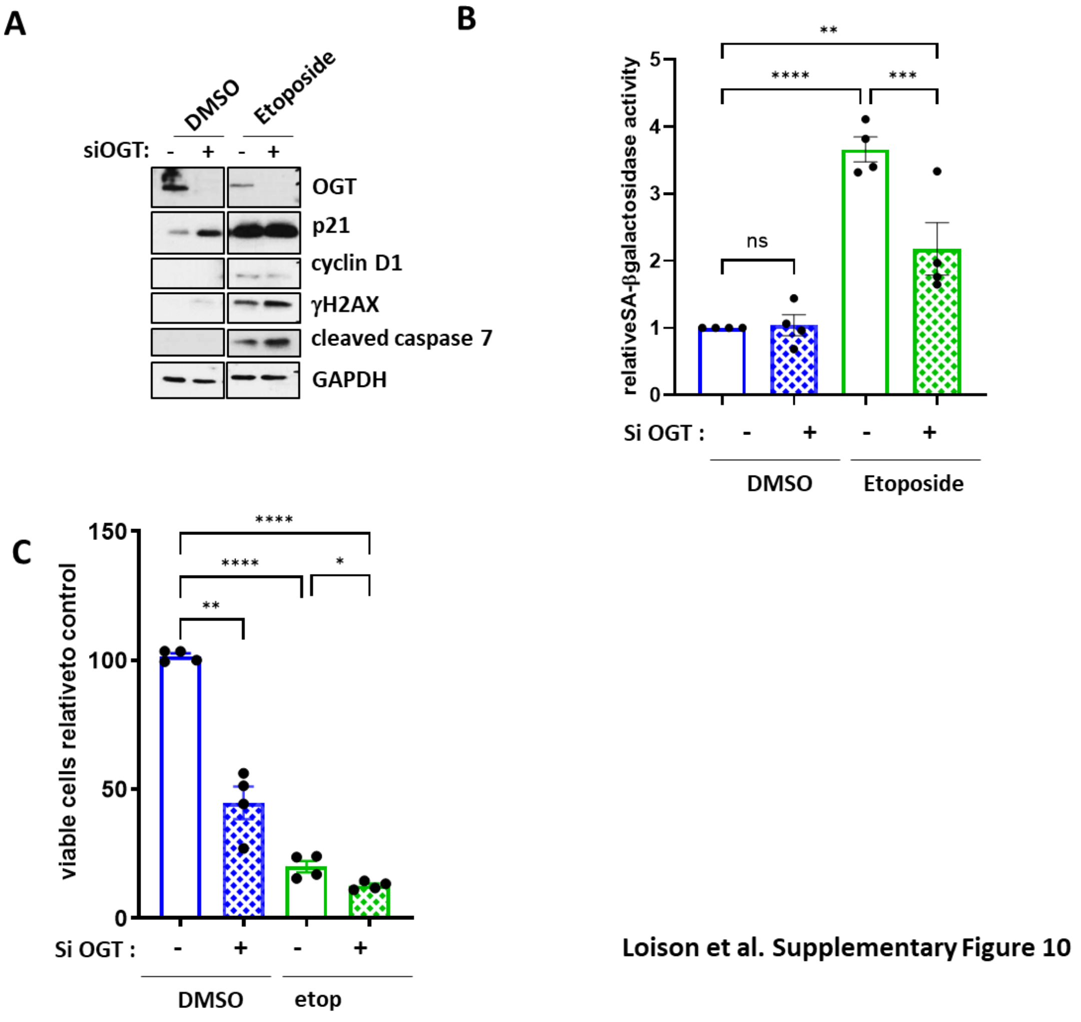


HCT116 cells were transfected with a siRNA targeting OGT (si OGT) or a non-target control siRNA (siCTRL). 24 hours later, cells were treated with 1 µM etoposide for 72 hours. **(A)** Western blot analysis was performed to assess the expression of the senescence markers p21 and cyclin D1, the DNA damage marker γH2AX and the apoptosis marker cleaved-caspase 7. The efficiency of siRNA was also confirmed by evaluating OGT loss of expression. GAPDH

was used as a loading control. Results shown are representative of four independent experiments. **(B)** Quantitative fluorimetric determination of the SA-β-Galactosidase activity in the different experimental conditions. RFU: Relative Florescence Unit. Results represent the individual values and mean +/- SEM of four independent experiments (n=4) (ns: non-significant, **P<0.01, ***P<0.001, ****P<0.0001, ordinary one-way ANOVA with Fisher's LSD multiple comparison tests). **(C)**: quantification of cell viability through MTT assay from four independent experiments (n=4) (individual values and mean +/- SEM, ns: non-significant, *P <0.05, **P<0.01, ****P<0.0001, ordinary one-way ANOVA with Fisher's LSD multiple comparison tests).

**Supplementary Figure S11: Pharmacological inhibition of OGT combined with low-dose SN38 treatment causes LS174T cells to switch from senescence to apoptosis in a dose dependent-manner.**

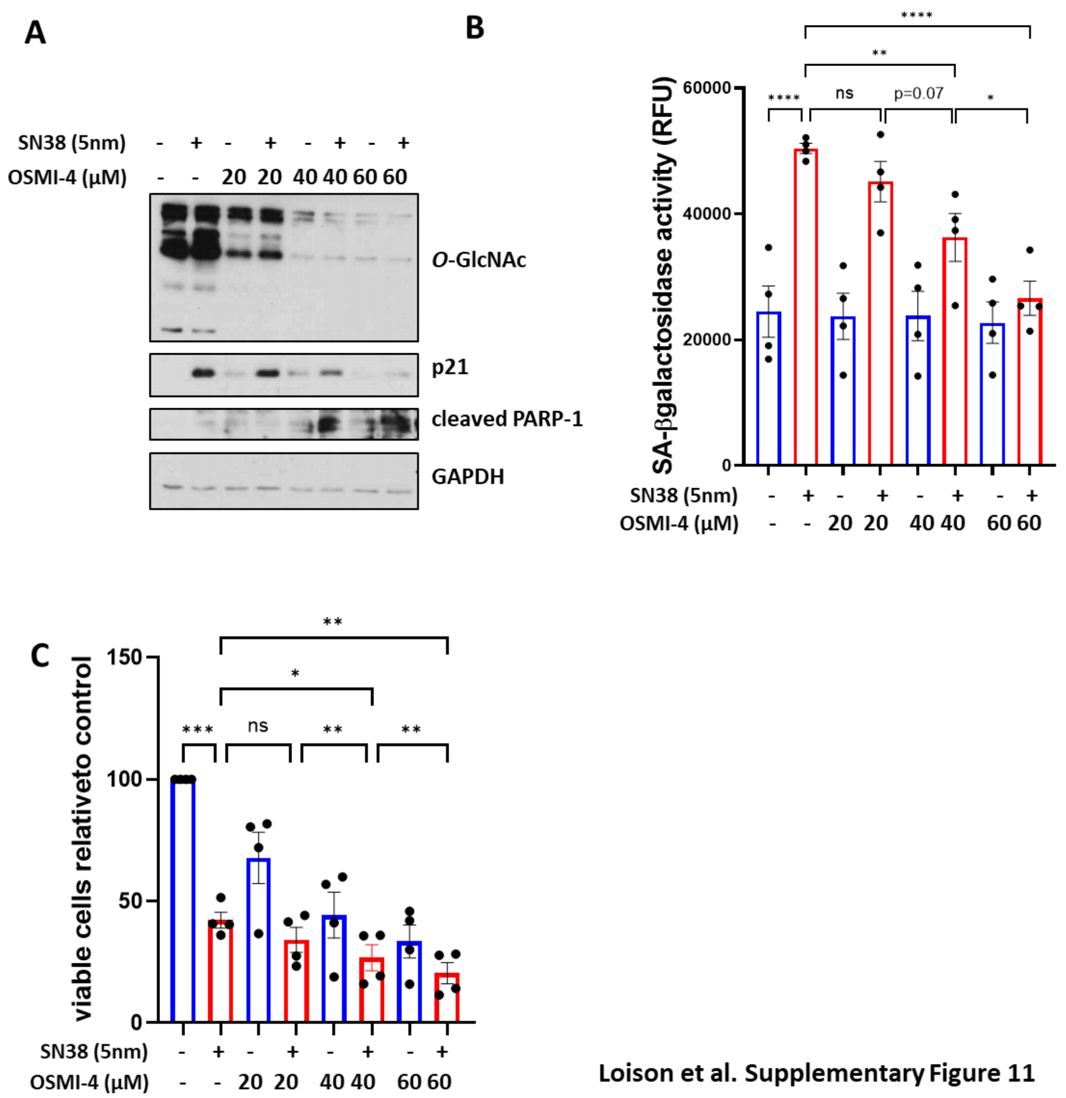


LS174T cells were treated with increasing concentrations of the OGT inhibitor OSMI-4 ranging from 20 to 60 µM. 24 hours later, the cells were treated with 1 µM etoposide for 72 hours. **(A)** Western blot analysis was performed to assess the expression of the senescence markers p21 and the apoptosis marker cleaved-PARP1. The treatment efficiency was also confirmed by

evaluating *O*-GlcNAcylation levels. GAPDH was used as a loading control. Results shown are representative of four independent experiments (n=4). **(B)** Quantitative fluorimetric determination of the SA-β-Galactosidase activity in the different experimental conditions. RFU: Relative Florescence Unit. Results represent the individual values and mean +/- SEM of four independent experiments (n=4) (ns: non-significant, *P<0.05, **P<0.01, ****P<0.0001, ordinary one-way ANOVA with Fisher's LSD multiple comparison tests). **(C)**: quantification of cell viability through MTT assay from four independent experiments (n=4) (individual values and mean +/- SEM, ns: non-significant, *P <0.05, **P<0.01, ***P<0.001, ordinary one-way ANOVA with Fisher's LSD multiple comparison tests).

**Supplementary Figure S12: The OGT inhibitor OSMI-4 does not enhance the cytotoxicity of SN38 in healthy colon organoïds.**

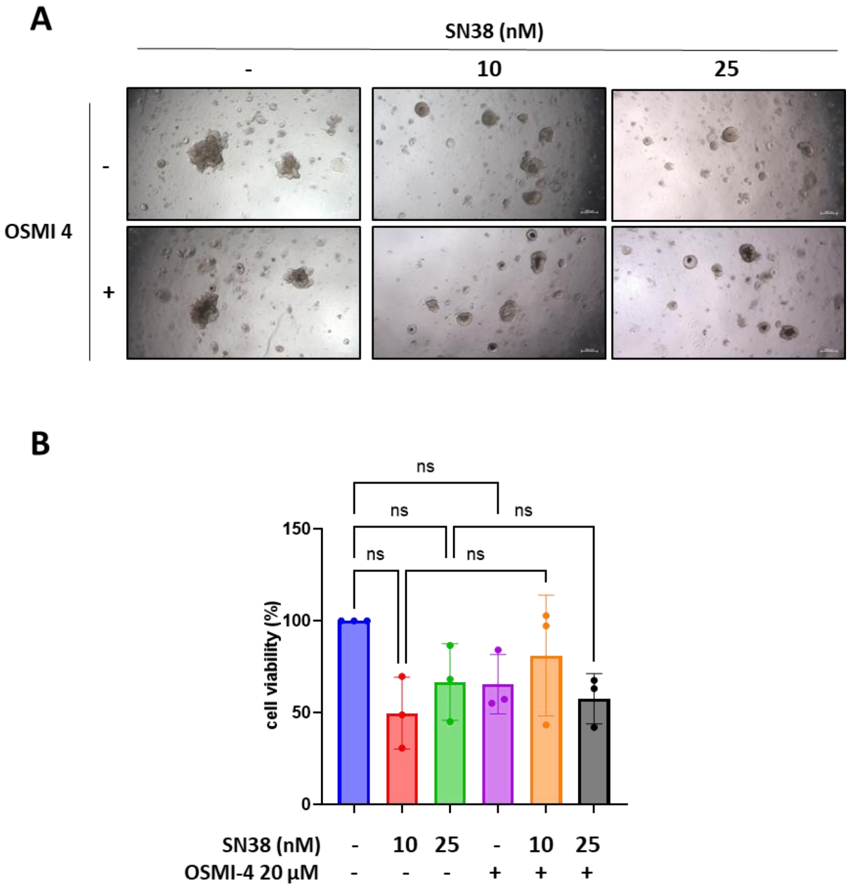


Organoids generated from healthy colon tissue of a 63-year-old female were allowed to form before being treated either with 10 nM or 25 nM of SN38 alone or in combination with 20 µM OSMI-4 for five days. **(A)** Representative brightfield images of the organoïds in the different experimental conditions. Scale bar = 200 µM. **(B)** Evaluation of cell viability through cell-titer glo® assay. Values represent the mean +/- SD of three independent experiments (n=3) (ns: non-significant, ordinary one-way ANOVA with Tukey’s multiple comparisons tests).

## Supplementary Table S1: List of the RT-qPCR primers used in this study

| Gene | Forward Primer | Reverse Primer |
|---|---|---|
| GFAT1 | TGCCGGCGTTTGATTCTTAT | ATCACAGGCAACTCAGTCAGCT |
| GFAT2 | CAGTTGGAAGGTGCATTCGC | GTATTTGCTCCGGACTCCGAT |
| OGT | TGGCTTCAGGAAGGCTATTG | CAAGTCTTTTGGATGTTCATATGG |
| OGA | ATGTTTTGGCGAGAGATGTATTC | AGGTGAGATCGCATAGATGAACT |
| p21 | GACTCTCAGGGTCGAAAACG | GGCTTCCTCTTGGAGAAGATCA |
| Cyclin D1 | CATCTACACCGACAACTCCATCC | TGTTCAATGAAATCGTGCGG |
| EZH2 | TGCAGTTGCTTCAGTACCCATAAT | ATCCCCGTGTACTTTCCCATCATAAT |
| EAR | GAGGCTGAGGCAGGAGAATCG | GTCGCCCAGGCTGGAGTG |
