# Supplementary material for: O-GlcNAcylation inhibition redirects the response of colon cancer cells to chemotherapy from senescence to apoptosis
Source: Cell Death Dis. 2024 Oct 19;15(10):762. doi: 10.1038/s41419-024-07131-5 (PMC11490504; doi:10.1038/s41419-024-07131-5)

Figure 1B

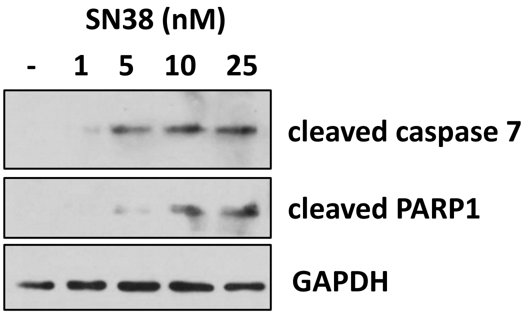

cleaved caspase 7

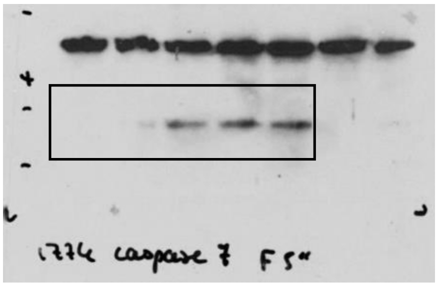

cleaved PARP1

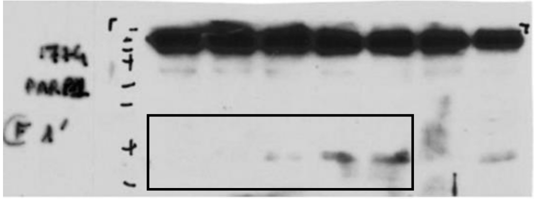

GAPDH

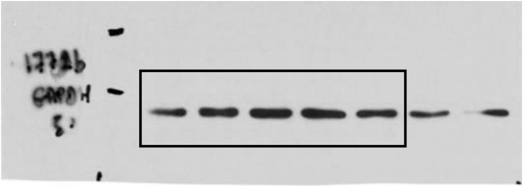

Figure 1D

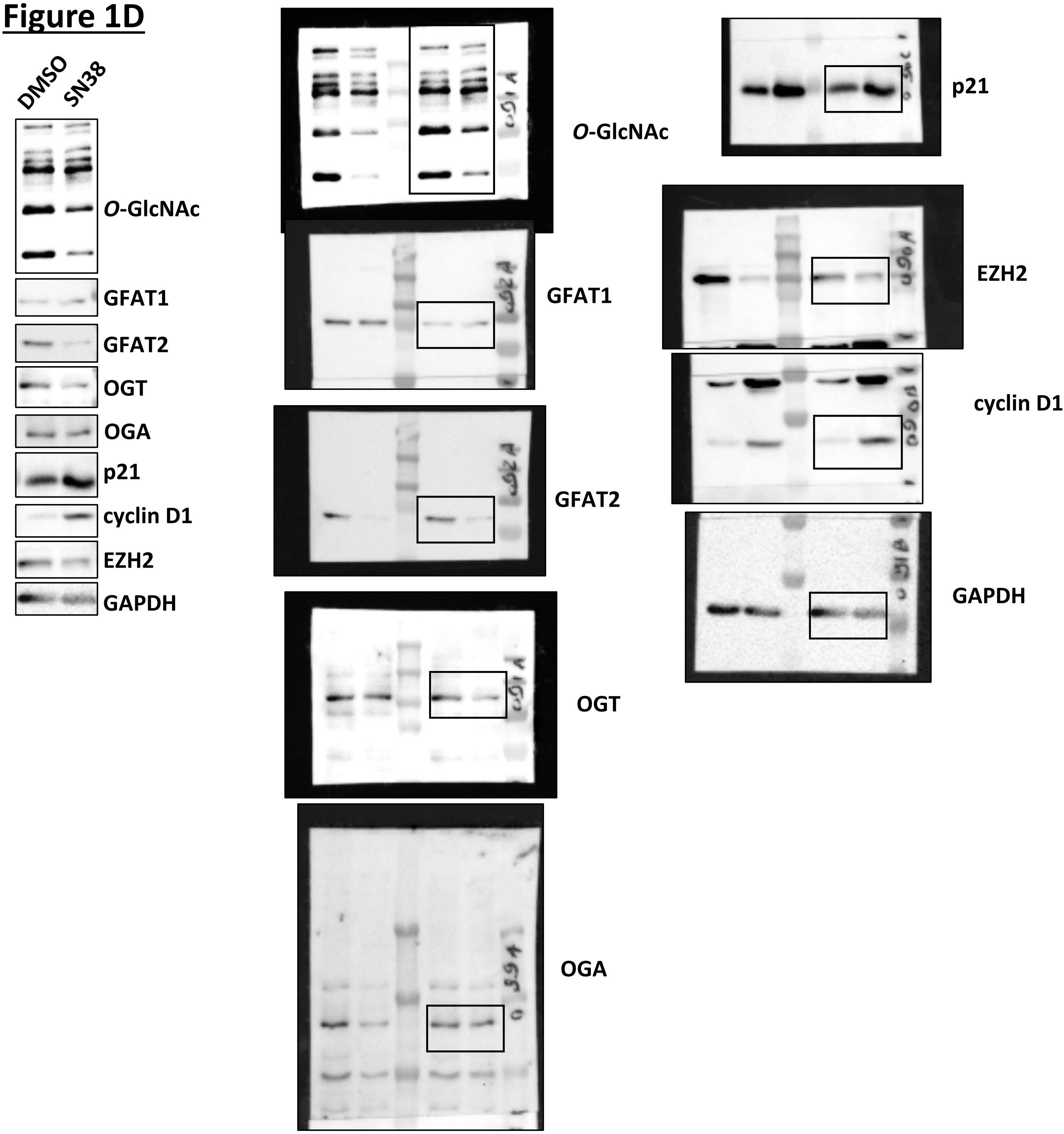

DMSO
SN38
O-GlcNAc
GFAT1
GFAT2
OGT
OGA
p21
cyclin D1
EZH2
GAPDH
O-GlcNAc
GFAT1
GFAT2
OGT
OGA
p21
EZH2
cyclin D1
GAPDH

Figure 2A

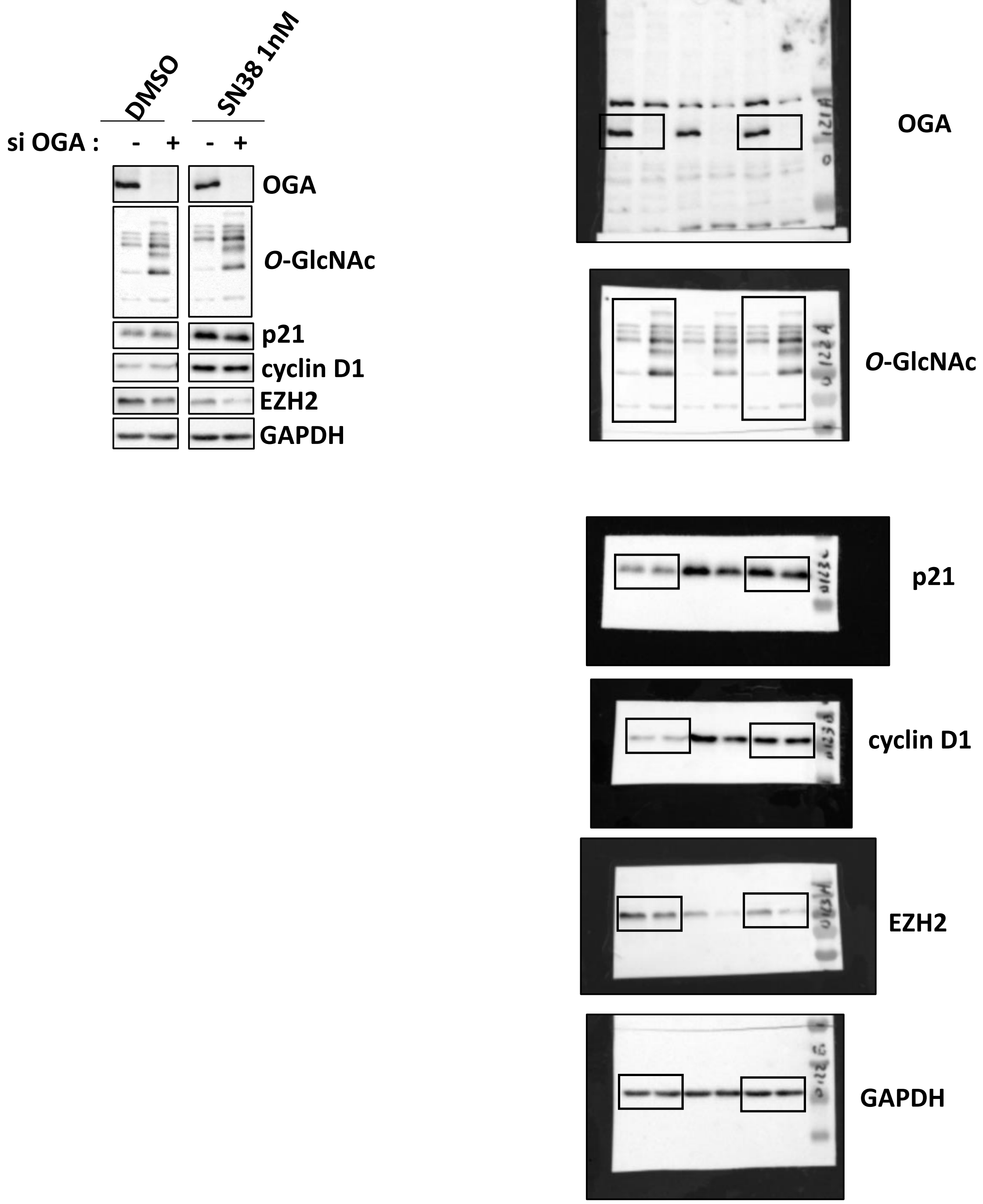
DMSO
SN38 1nM
si OGA : - + - +
OGA
O-GlcNAc
p21
cyclin D1
EZH2
GAPDH
OGA
O-GlcNAc
p21
cyclin D1
EZH2
GAPDH

Figure 3A

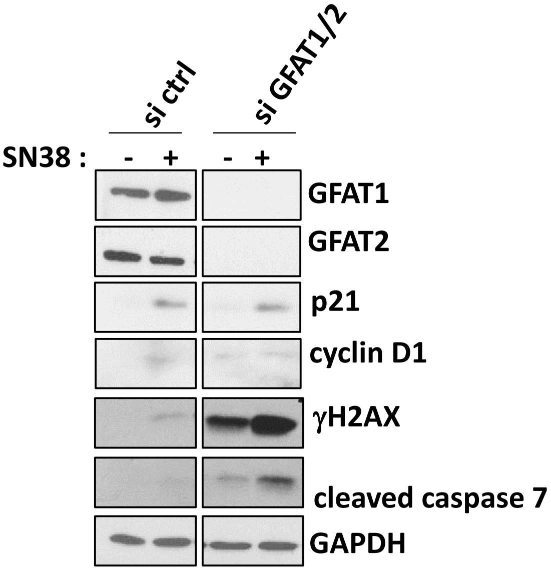

si ctrl
si GFAT1/2
SN38 :
-
+
-
+
GFAT1
GFAT2
p21
cyclin D1
γH2AX
cleaved caspase 7
GAPDH

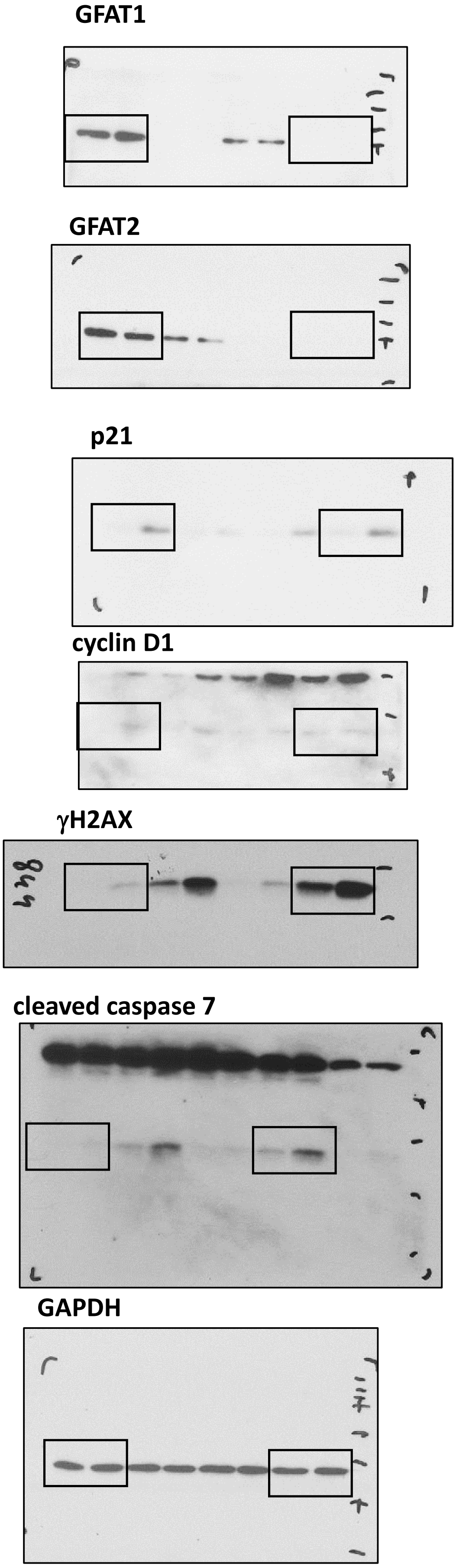

GFAT1
GFAT2
p21
cyclin D1
γH2AX
cleaved caspase 7
GAPDH

Figure 4A

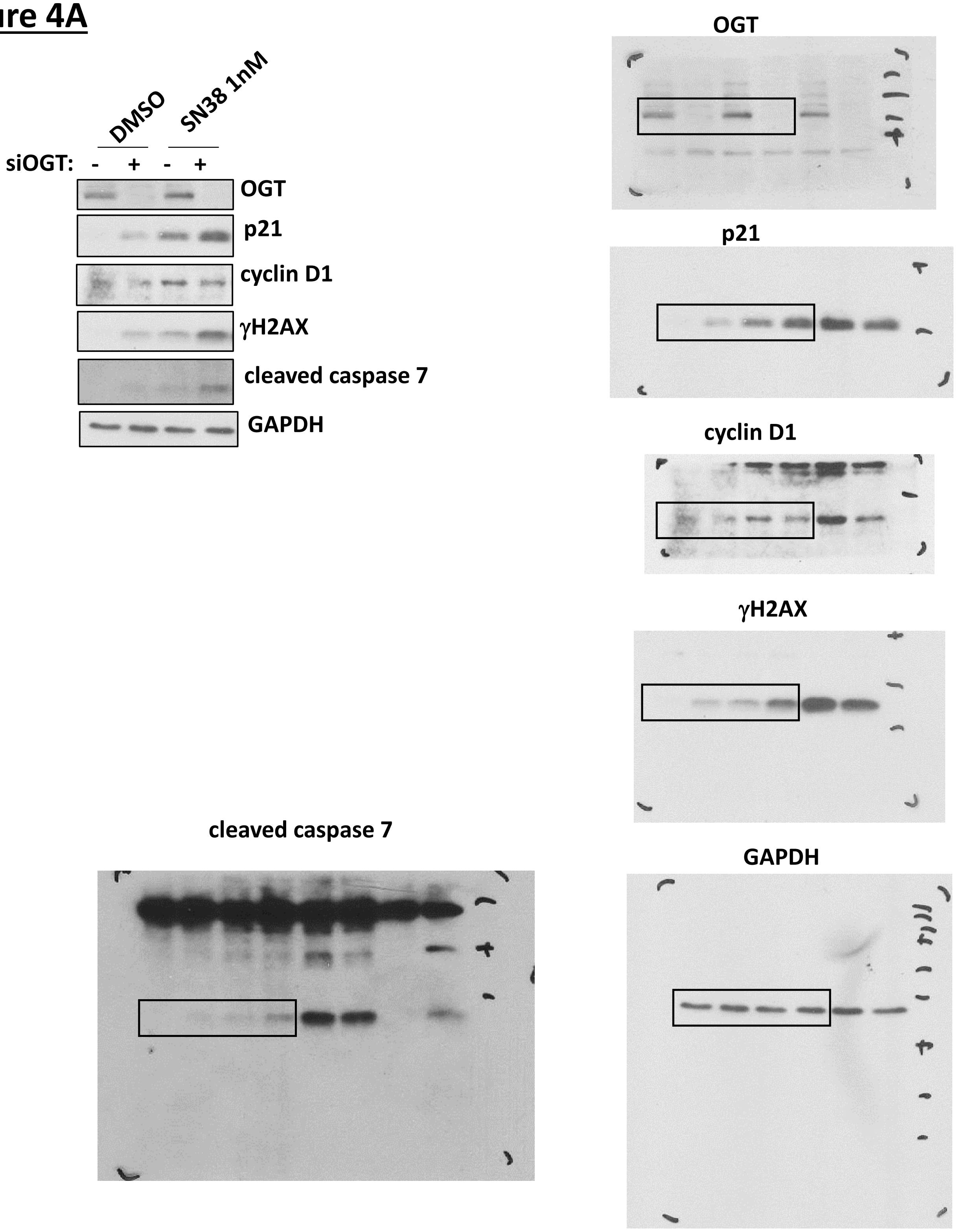
DMSO
SN38 1nM
siOGT: - + - +
OGT
p21
cyclin D1
γH2AX
cleaved caspase 7
GAPDH
OGT
p21
cyclin D1
γH2AX
cleaved caspase 7
GAPDH

Figure 5A

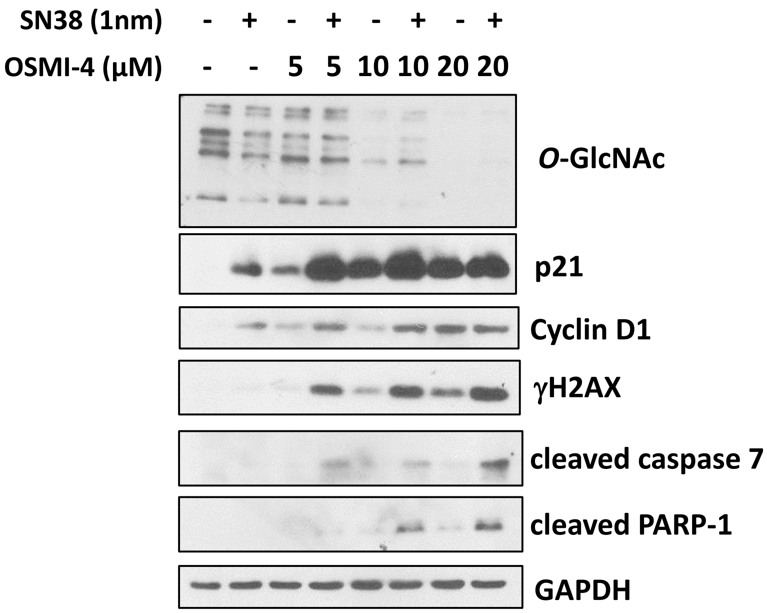

O-GlcNAc

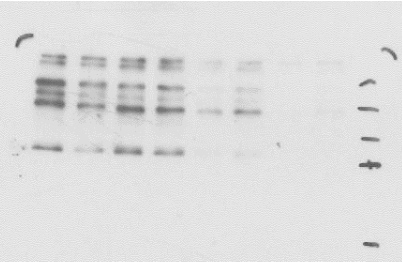

p21

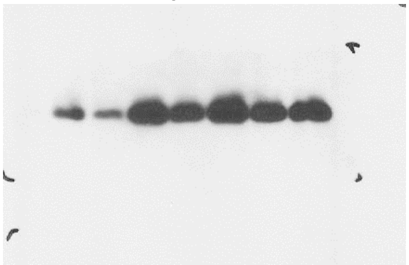

Cyclin D1

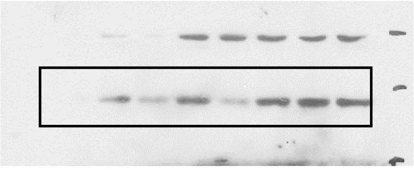

γH2AX

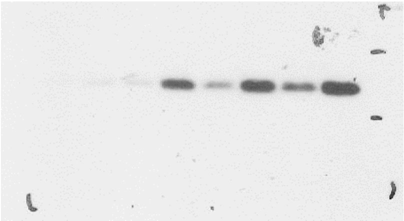

GAPDH

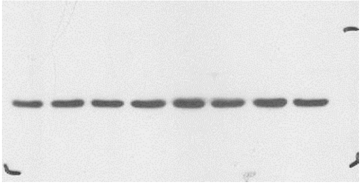

cleaved caspase 7

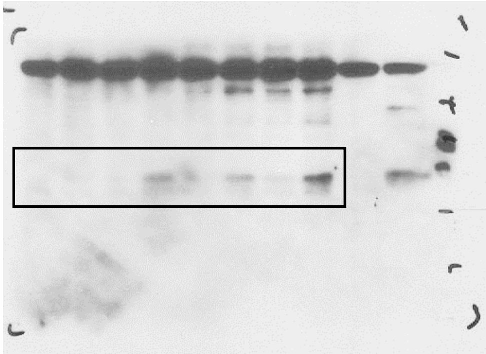

cleaved PARP-1

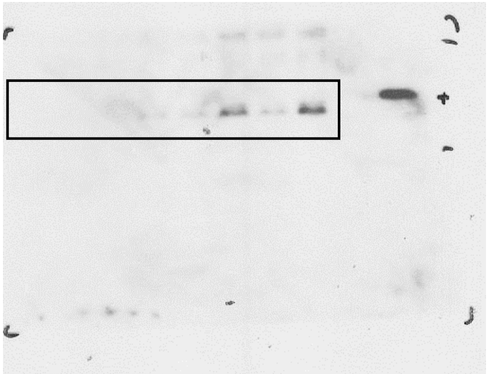

Supplementary figure S1B

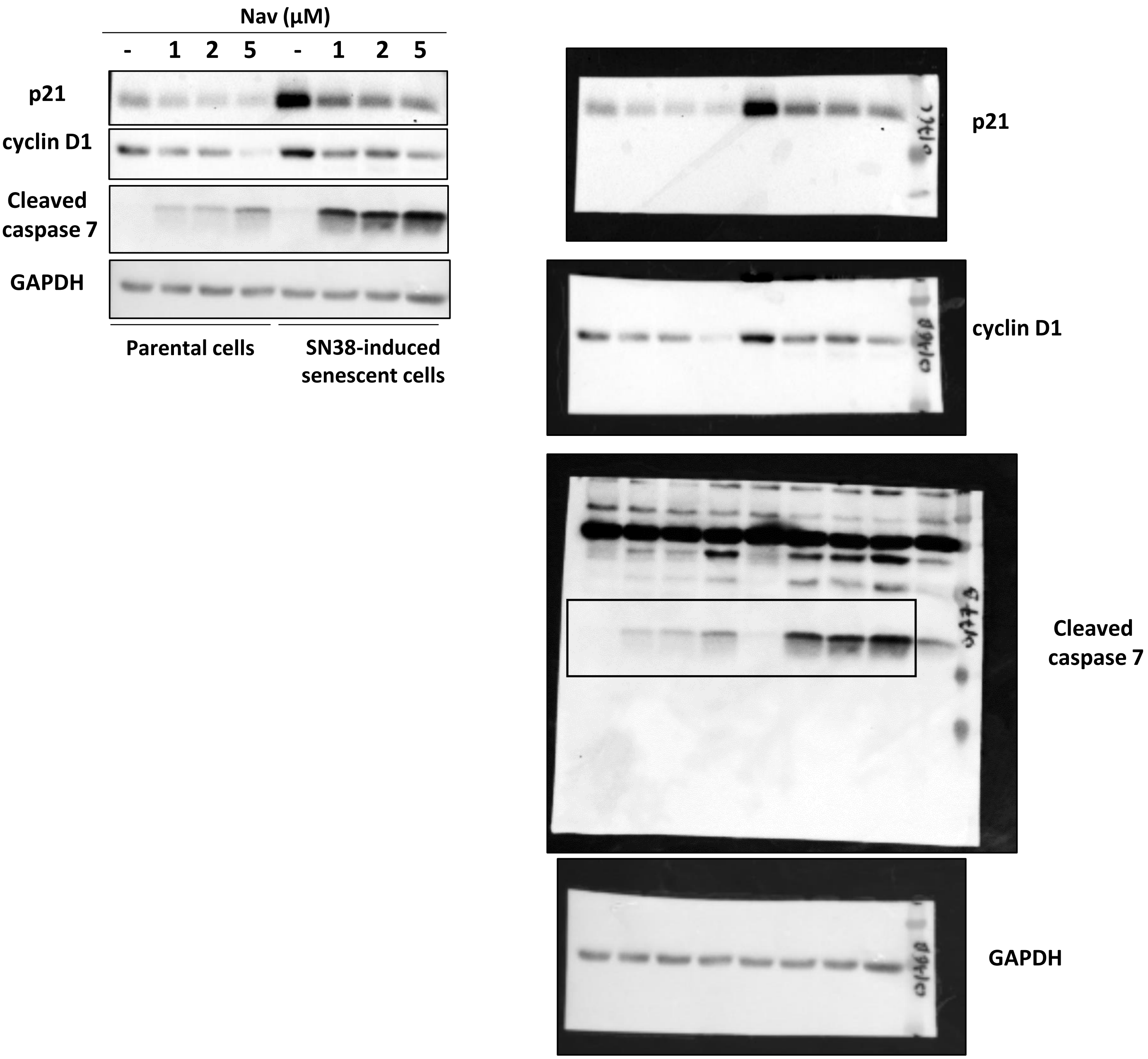

Supplementary figure S2A

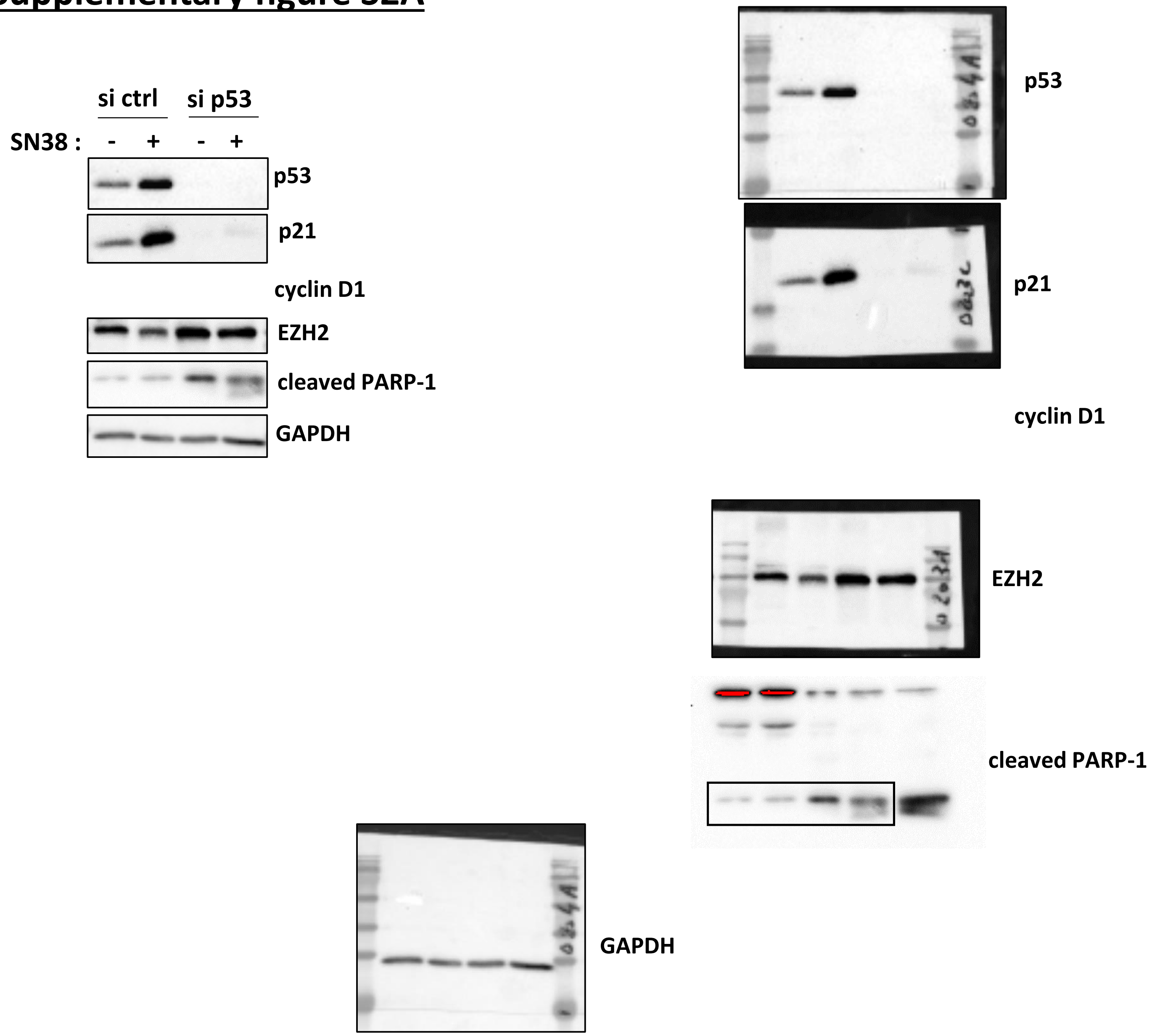

Supplementary figure S2C

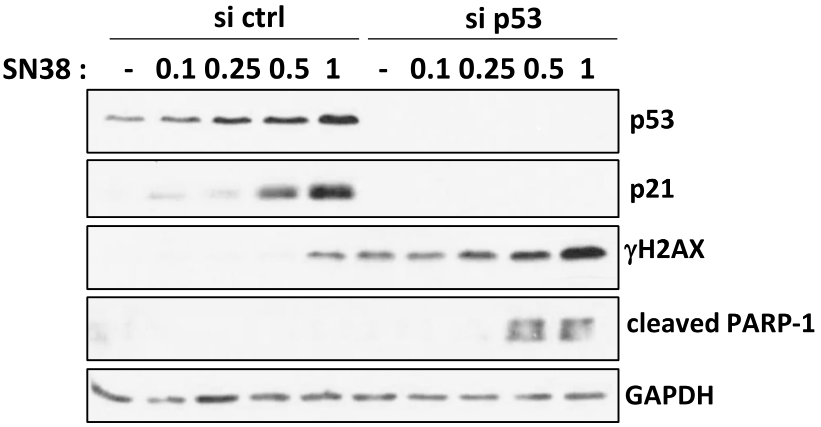

p53

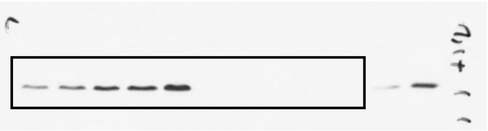

p21

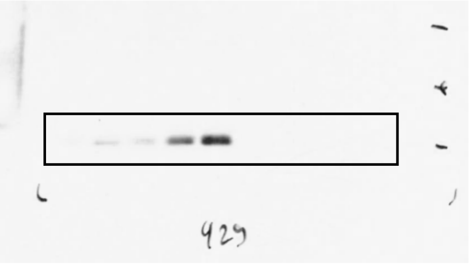

γH2AX

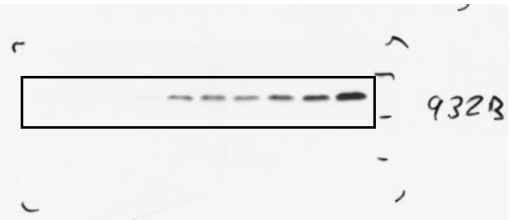

Cleaved PARP-1

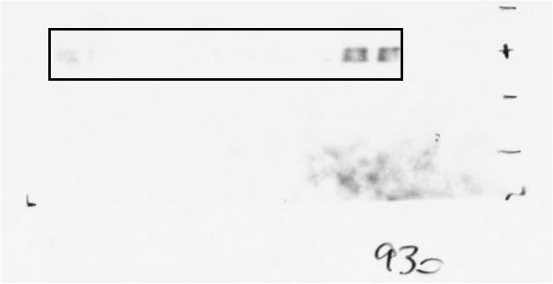

GAPDH

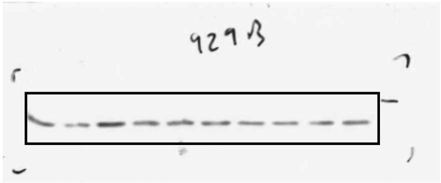

Supplementary figure S3B

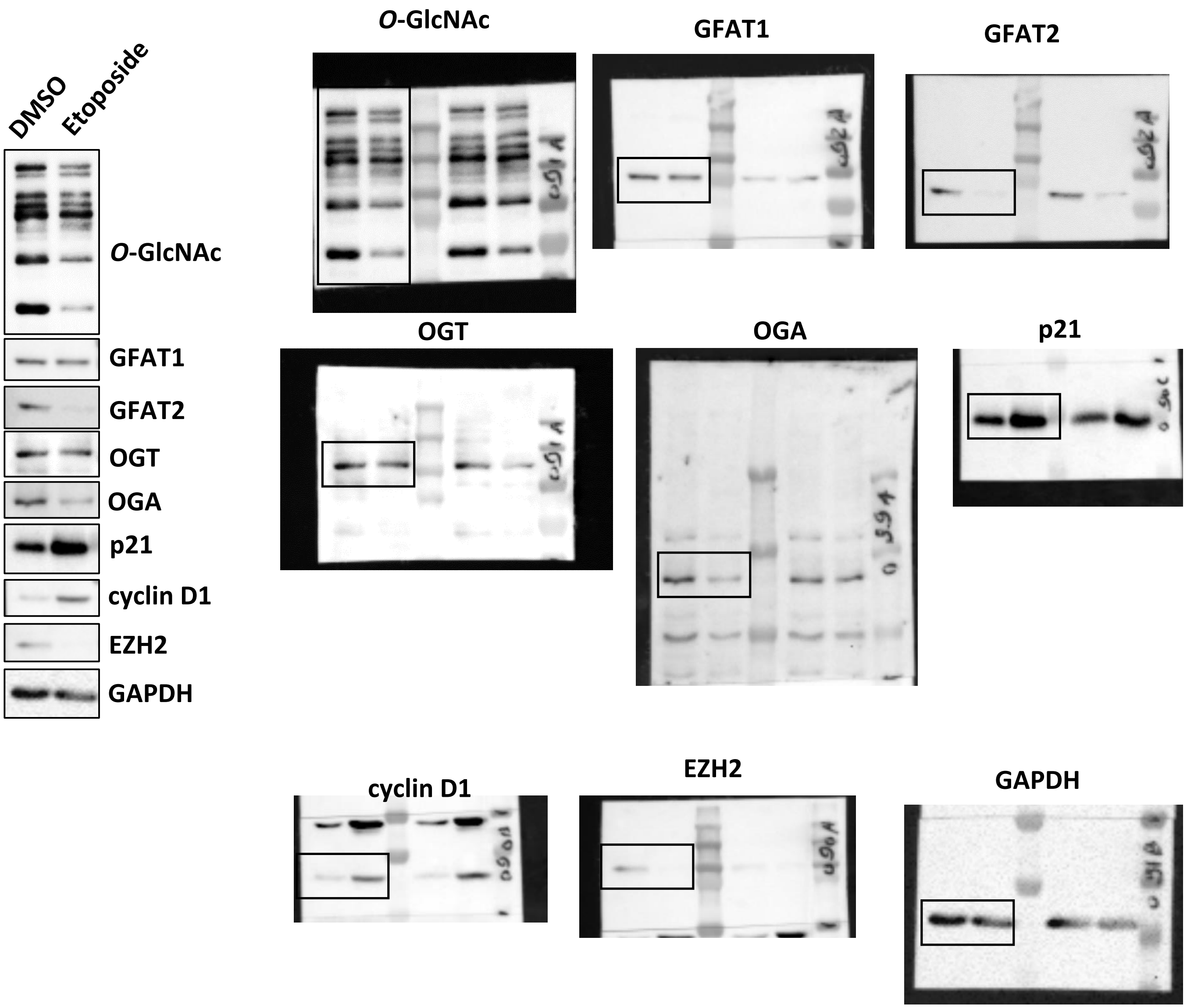

Supplementary figure S4A

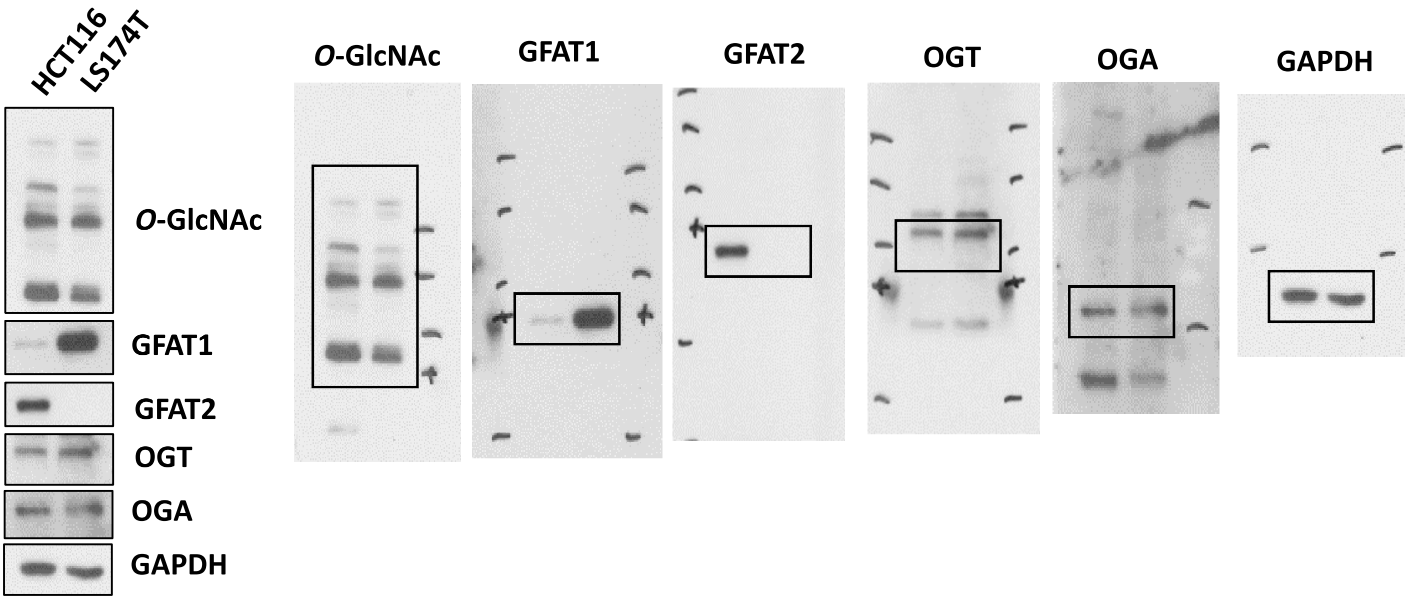

Supplementary figure S4C

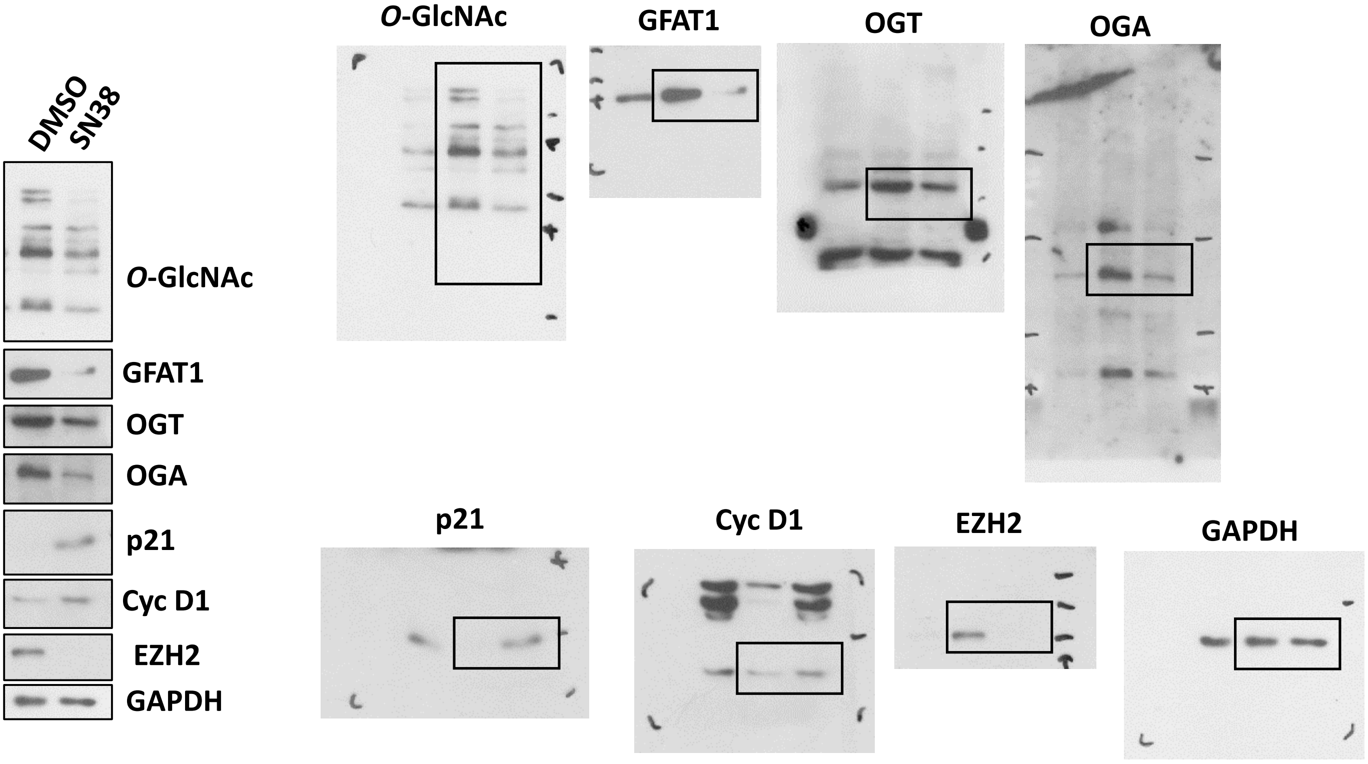

Supplementary figure S4C

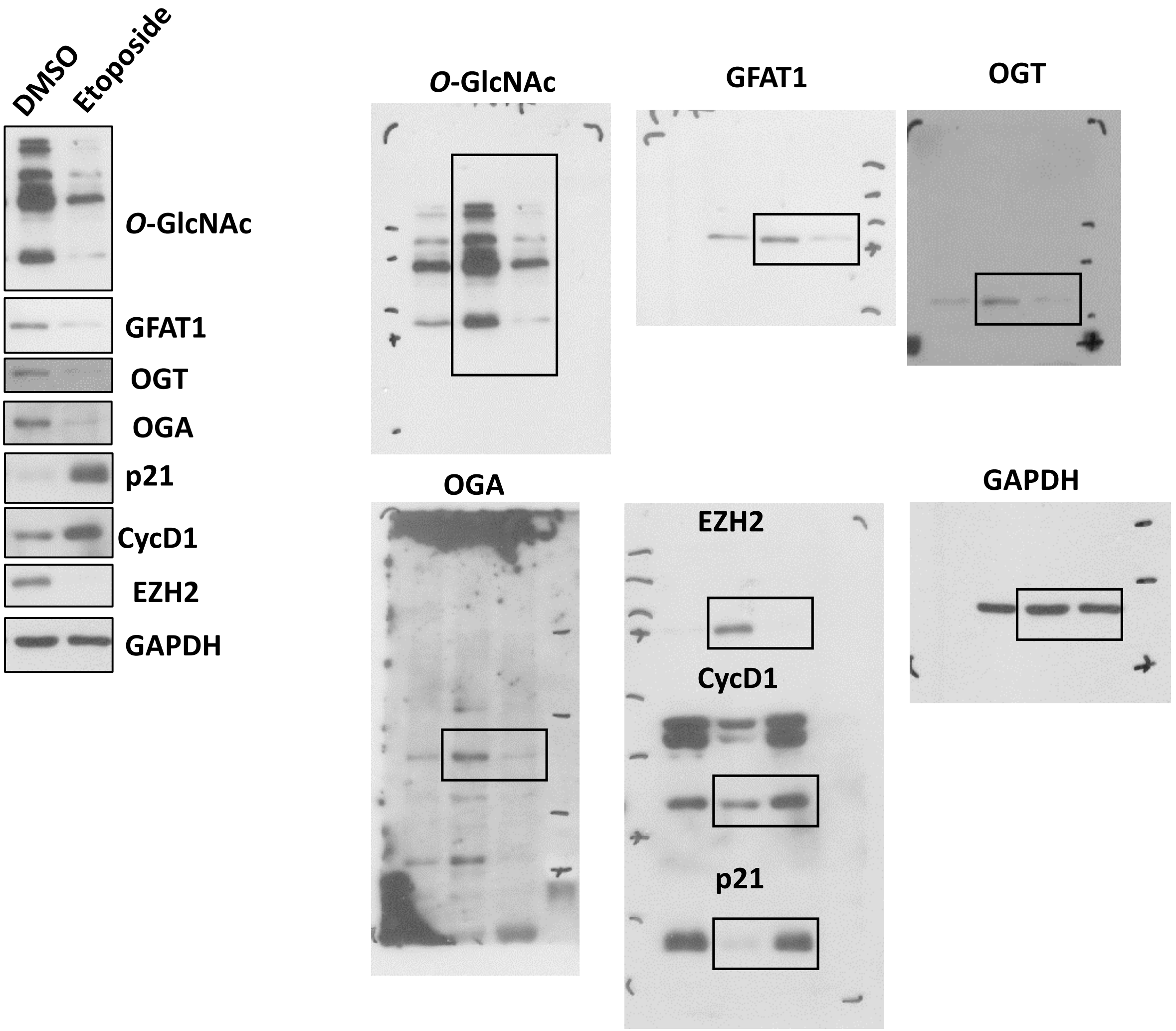

Supplementary figure S5A

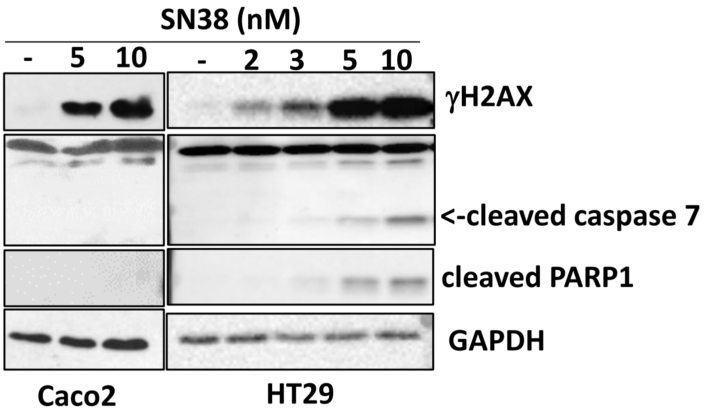

Caco2

γH2AX

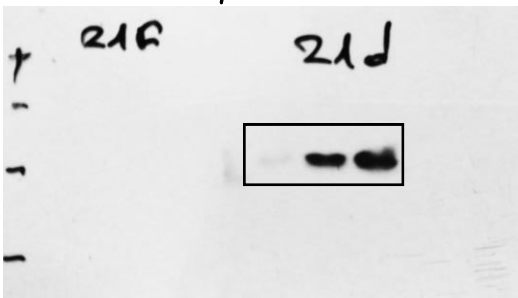

caspase 7

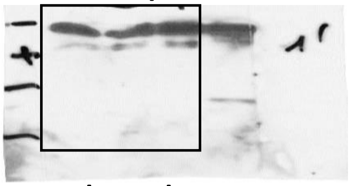

cleaved PARP1

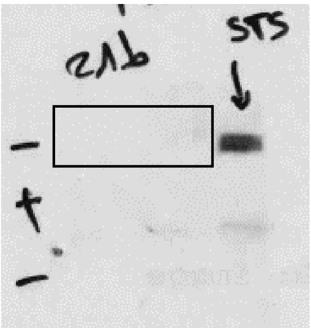

GAPDH

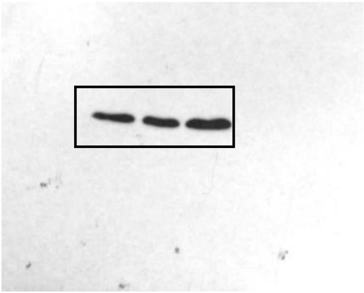

HT29

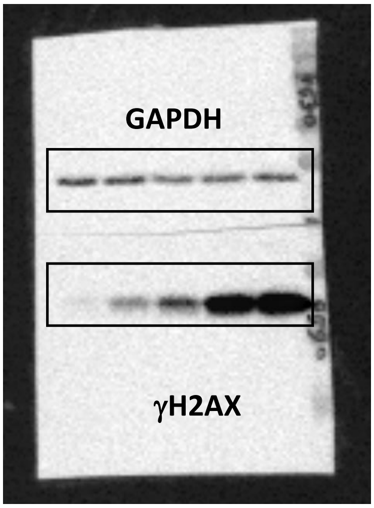

caspase 7

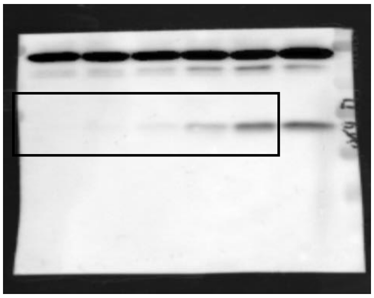

cleaved PARP1

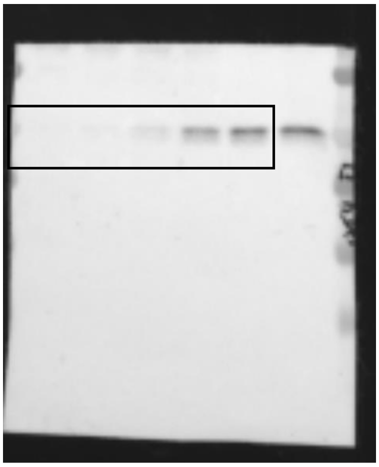

Supplementary figure S5D

D

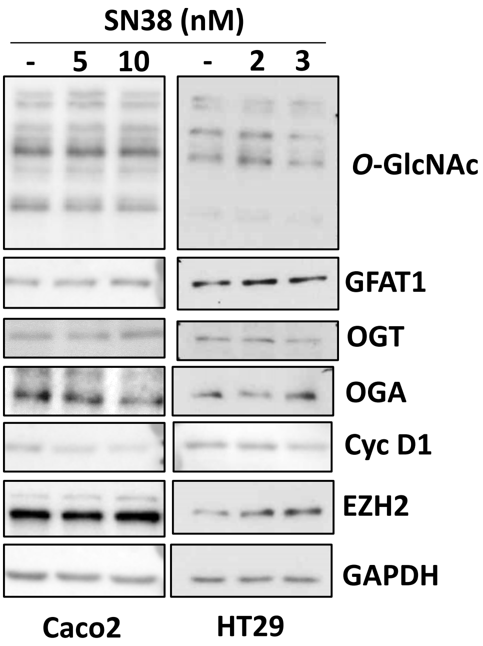

Caco2

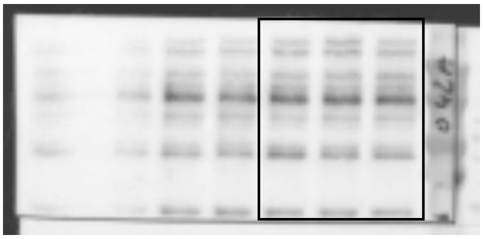

***O*-GlcNAc**

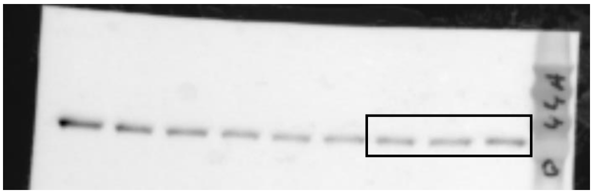

GFAT1

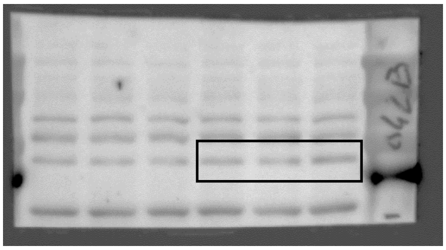

OGT

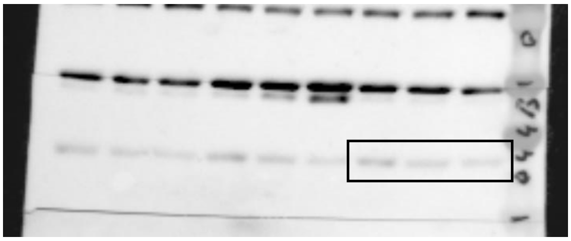

Cyc D1

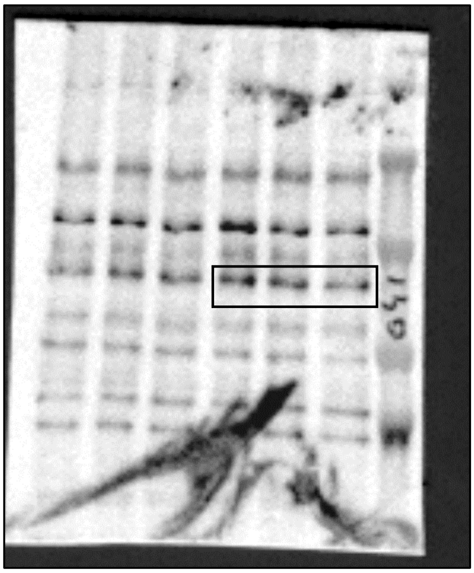

OGA

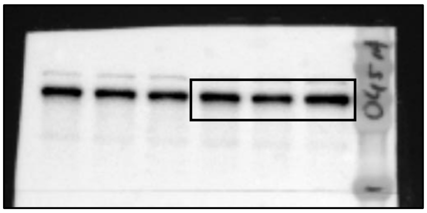

EZH2

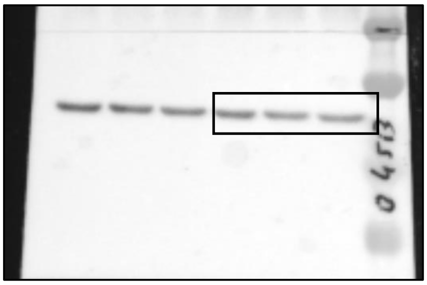

GAPDH

Supplementary figure S5D

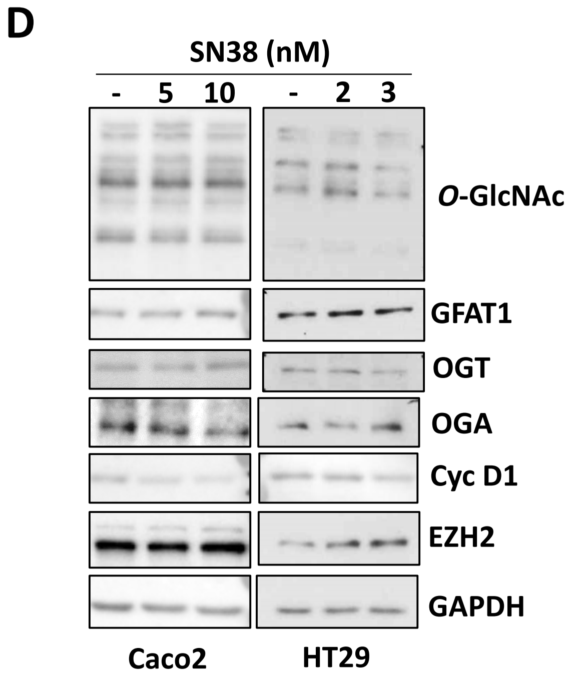

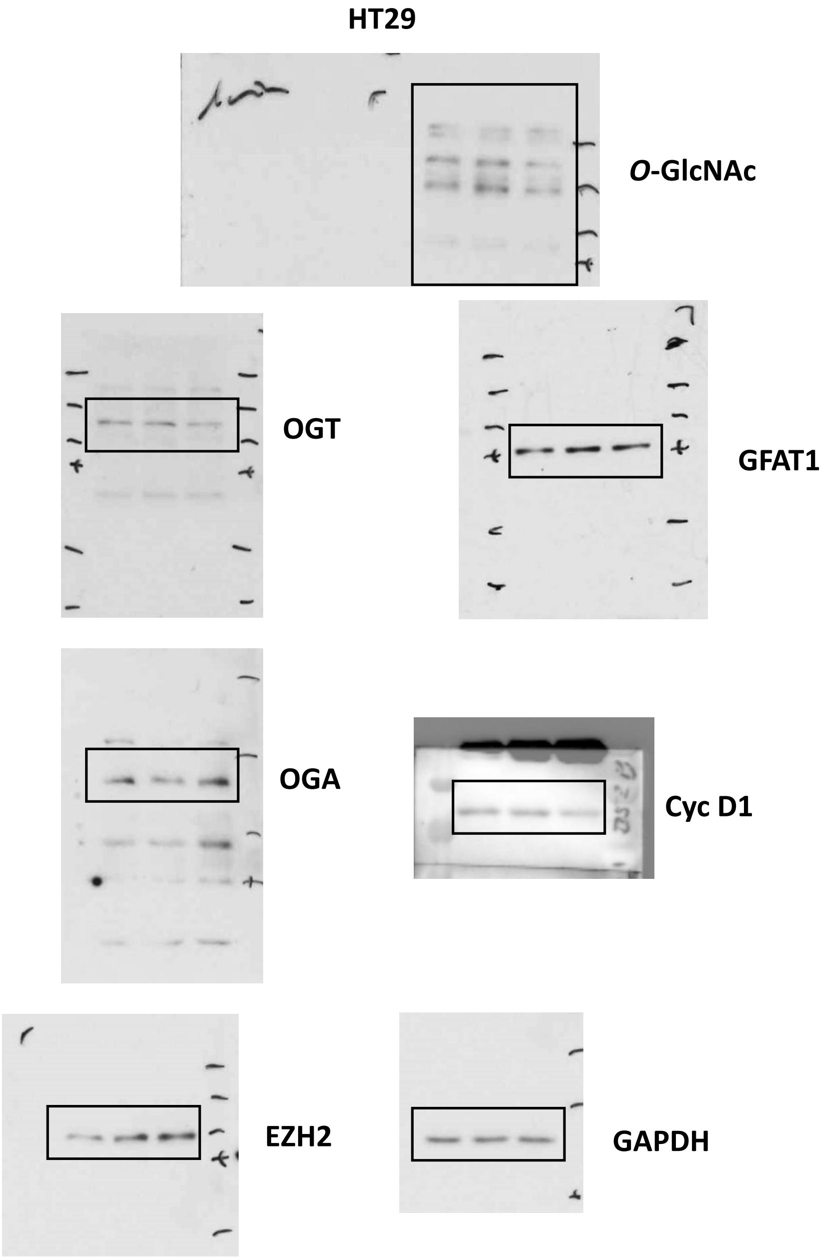

Supplementary figure S6A

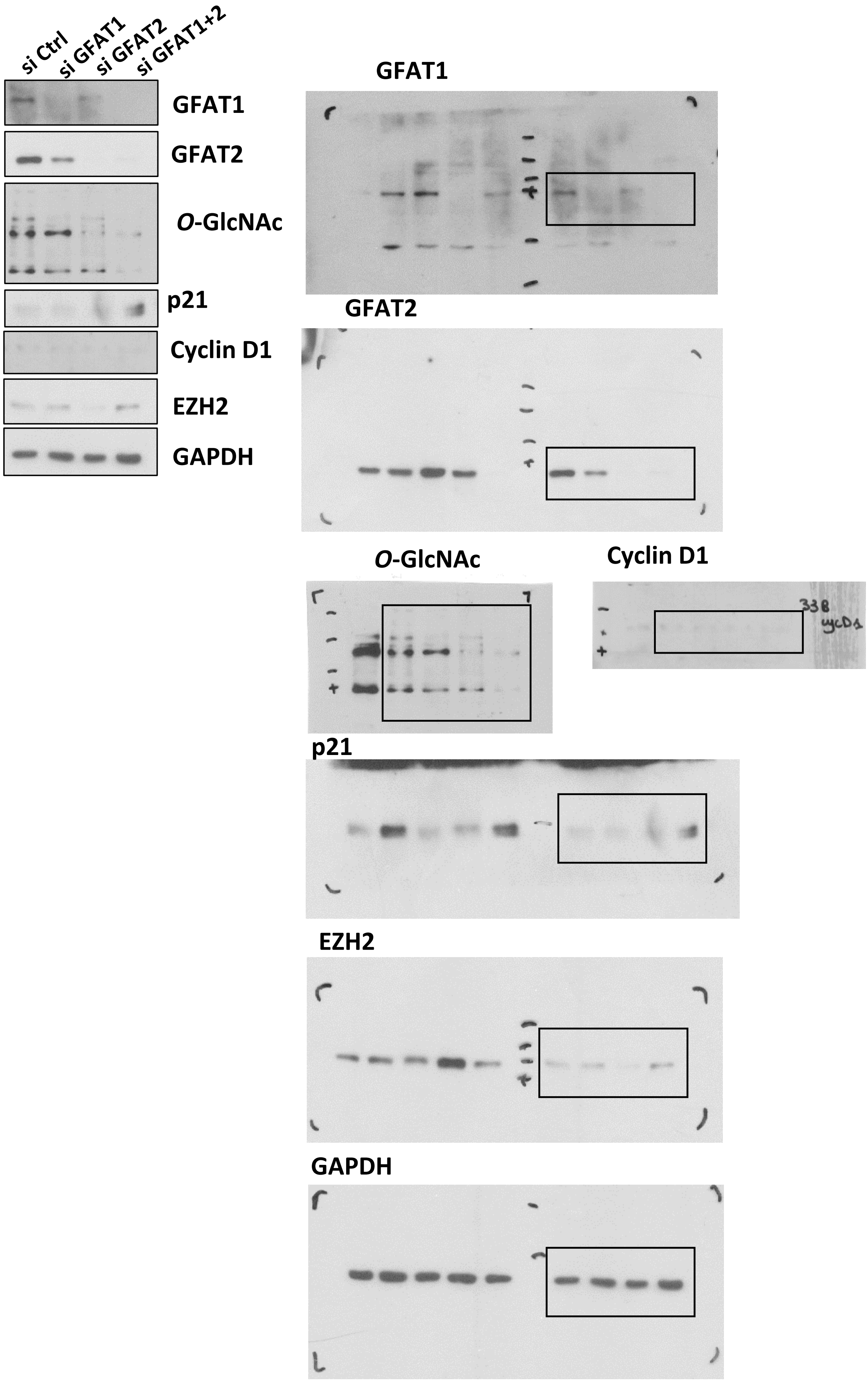

Supplementary figure S7A

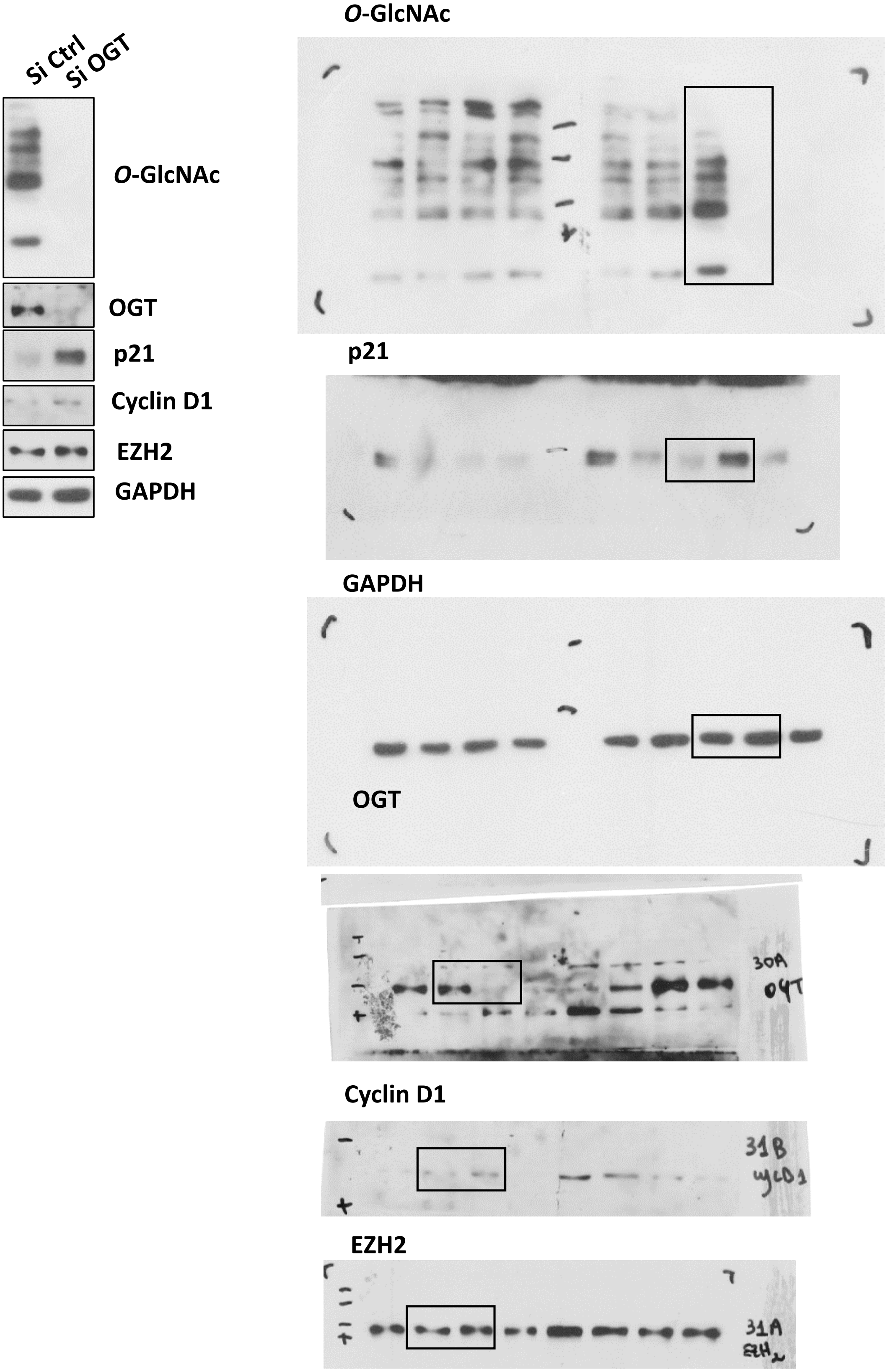

Supplementary figure S7C

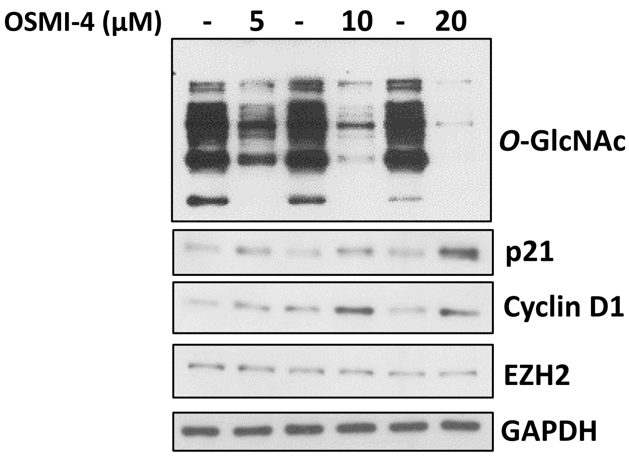

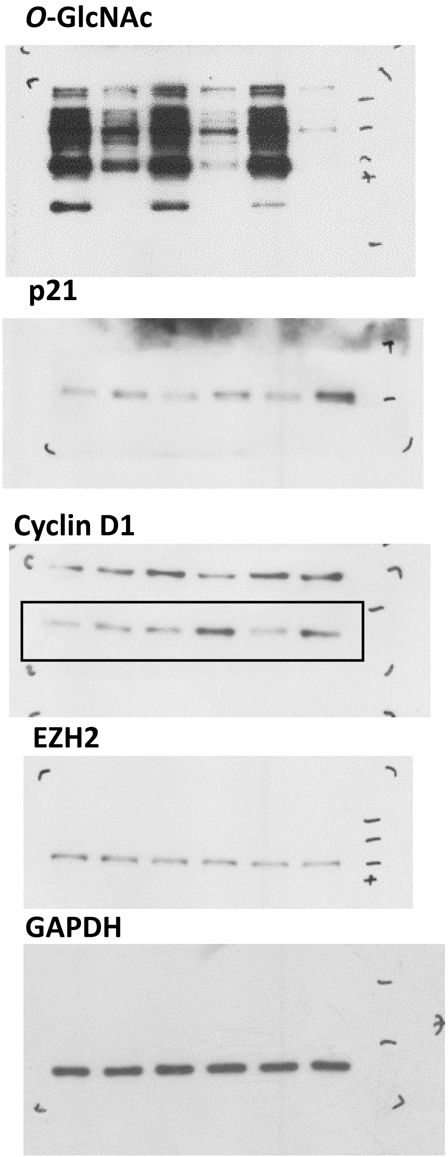

Supplementary figure S8A

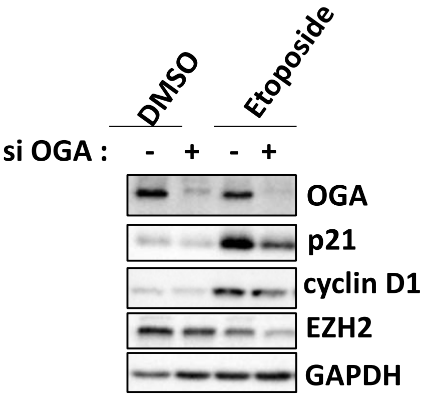

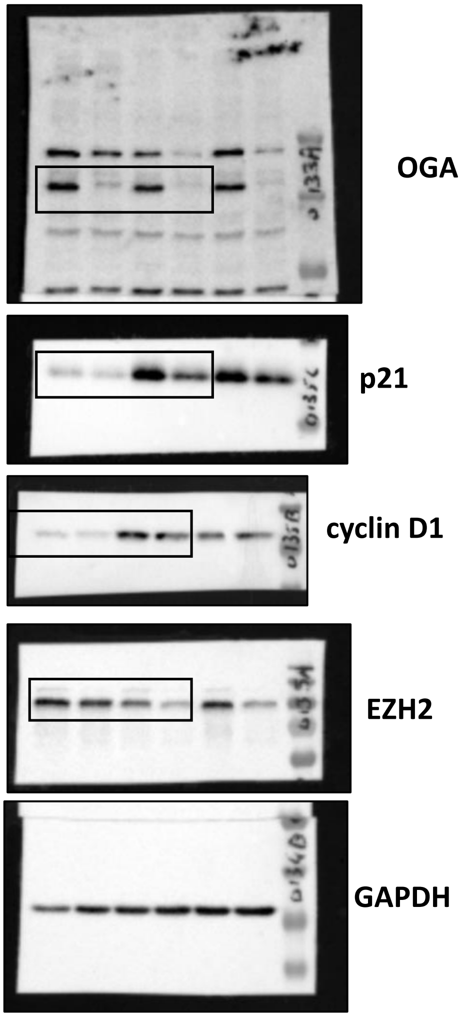

Supplementary figure S9A

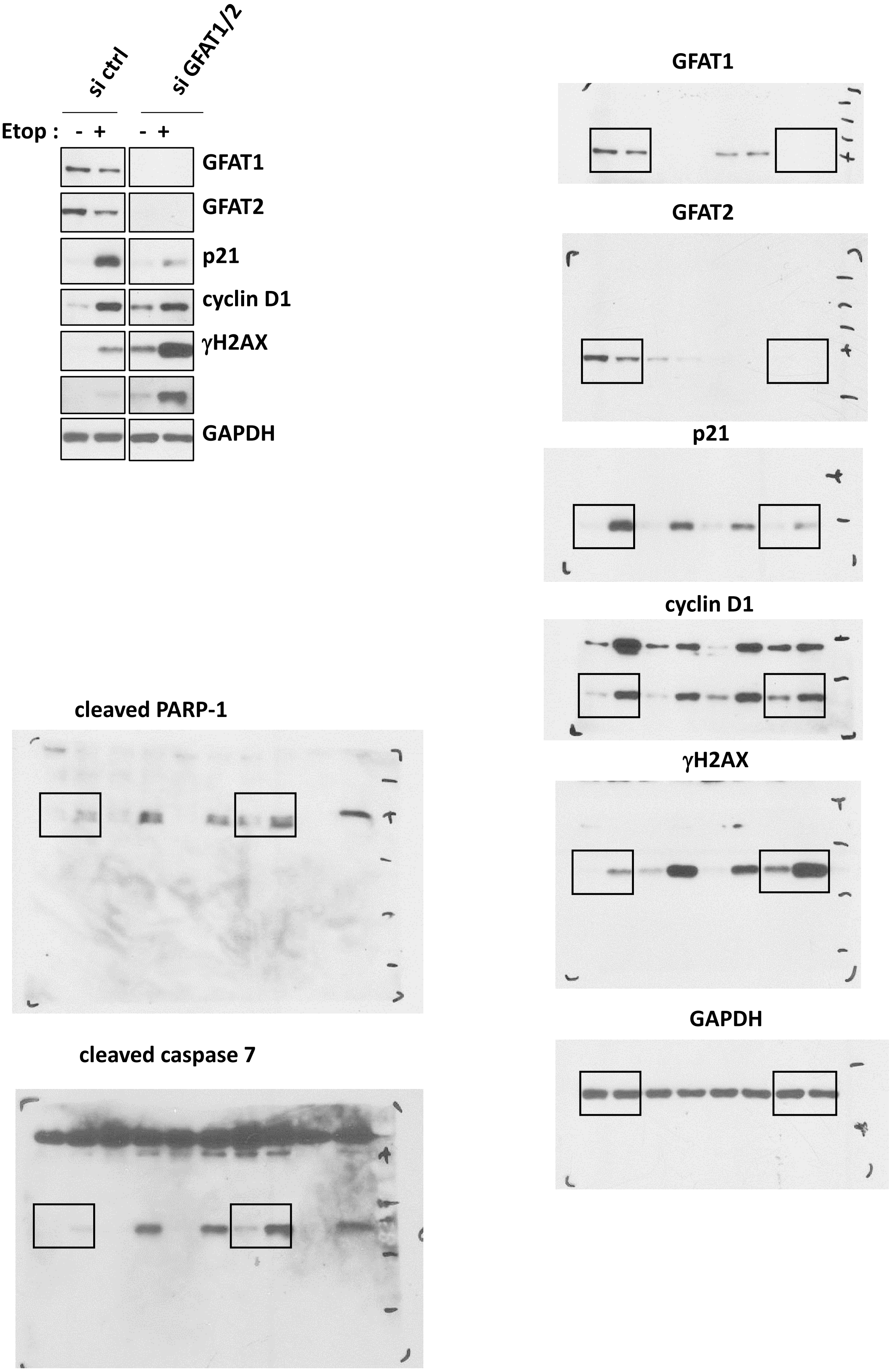

Supplementary figure S10A

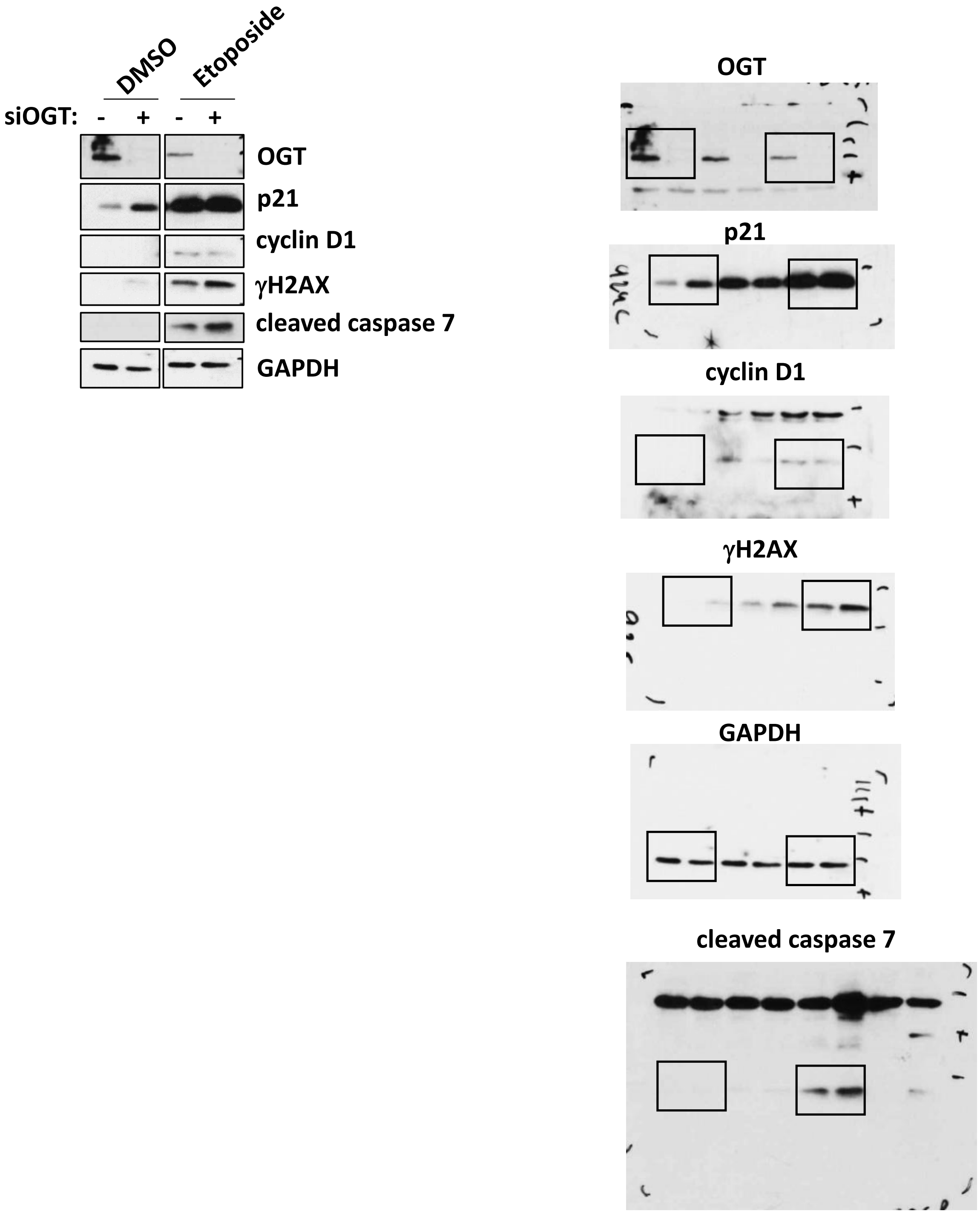

Supplementary figure S11A

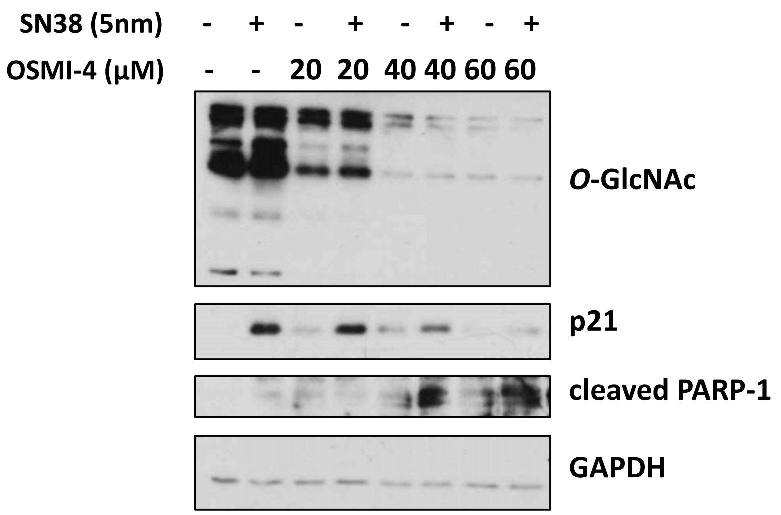

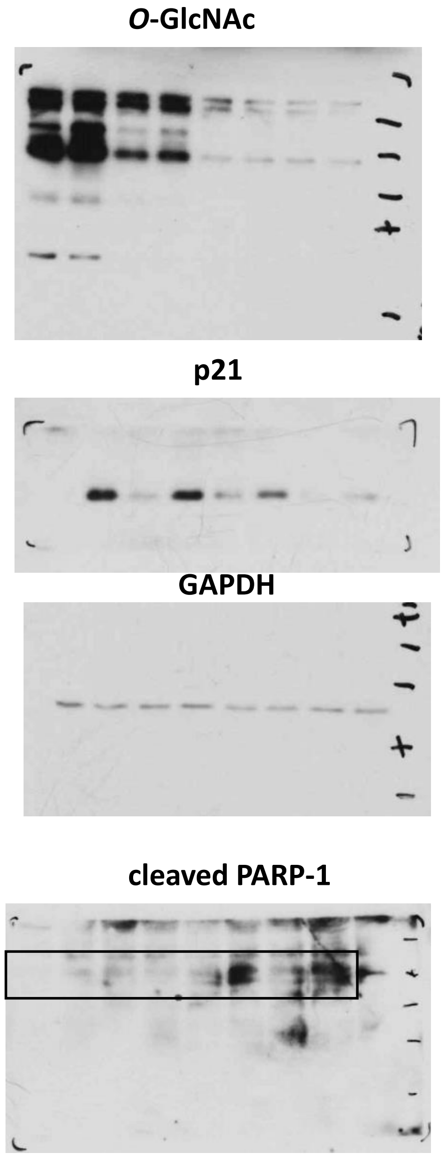

Supplement: Supplementary file 2 — Loison et al. 2024 non cropped blots [file 41419_2024_7131_MOESM2_ESM.pdf]
